# Rest phase snacking increases energy resorption and weight gain in male mice

**DOI:** 10.1016/j.molmet.2023.101691

**Published:** 2023-02-04

**Authors:** Kimberly Begemann, Isabel Heyde, Pia Witt, Julica Inderhees, Brinja Leinweber, Christiane E. Koch, Olaf Jöhren, Rebecca Oelkrug, Arkadiusz Liskiewicz, Timo D. Müller, Henrik Oster

**Affiliations:** 1 Institute of Neurobiology, University of Lübeck, Ratzeburger Allee 160, 23562 Lübeck, Germany; 2 Center of Brain, Behavior, and Metabolism, University of Lübeck, Ratzeburger Allee 160, 23562 Lübeck, Germany; 3 Bioanalytic Core Facility, University of Lübeck, Ratzeburger Allee 160, 23562 Lübeck, Germany; 4 Institute for Endocrinology and Diabetes, University of Lübeck, Ratzeburger Allee 160, 23562 Lübeck, Germany; 5 Institute for Diabetes and Obesity, Helmholtz Diabetes Center, Helmholtz Zentrum München, Neuherberg, Germany; 6 German Center for Diabetes Research (DZD), Neuherberg, Germany; 7 Department of Physiology, Faculty of Medical Sciences in Katowice, Medical University of Silesia, Katowice 40-752, Poland

**Keywords:** Body weight gain, Snacking, Circadian clock, Light–dark cycle, Energy intake, Energy resorption

## Abstract

**Objective:**

Snacking, *i.e*., the intake of small amounts of palatable food items, is a common behavior in modern societies, promoting overeating and obesity. Shifting food intake into the daily rest phase disrupts circadian rhythms and is also known to stimulate weight gain. We therefore hypothesized that chronic snacking in the inactive phase may promote body weight gain and that this effect is based on disruption of circadian clocks.

**Methods:**

Male mice were fed a daily chocolate snack either during their rest or their active phase and body weight development and metabolic parameters were investigated. Snacking experiments were repeated in constant darkness and in clock-deficient mutant mice to examine the role of external and internal time cues in mediating the metabolic effects of snacking.

**Results:**

Chronic snacking in the rest phase increased body weight gain and disrupted metabolic circadian rhythms in energy expenditure, body temperature, and locomotor activity. Additionally, these rest phase snacking mice assimilated more energy during the inactive phase. Body weight remained increased in rest phase snacking wildtype mice in constant darkness as well as in clock-deficient mutant mice under a regular light–dark cycle compared to mice snacking in the active phase. Weight gain effects were abolished in clock-deficient mice in constant darkness.

**Conclusions:**

Our data suggest that mistimed snacking increases energy resorption and promotes body weight gain. This effect requires a functional circadian clock at least under constant darkness conditions.

## Highlights

• Snack timing determines body weight gain and fat mass in male mice.
• Rest phase snacking disturbs daily rhythms in energy expenditure and activity.
• Rest phase snacking dampens intestinal clock gene expression rhythms.
• Rest phase snacking leads to higher energy resorption.
• Timed snack effects require a circadian clock or a rhythmic light–dark cycle.

## Graphical abstract

**Figure:**
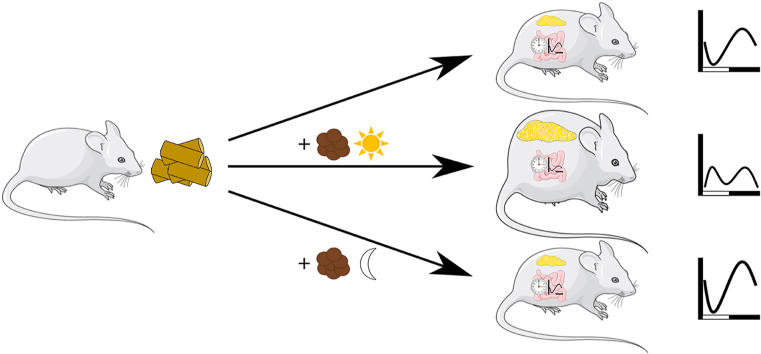

## 1 Introduction

Consuming small amounts of palatable food, *i.e.*, snacking, at various times of the day is a highly prevalent behavior in most modern societies. Chronic rest-phase food intake – especially of high-caloric items – promotes obesity and disrupts endogenous circadian rhythms [[1], [2], [3]]. Notably, humans and mice are more prone to hedonically driven eating behavior, the overconsumption of palatable food, during the late active/early inactive phase, *i.e.*, the morning in mice, the evening in humans [4,5]. While the effects of calorie-dense food items in promoting body weight gain are well documented, the metabolic impact of snack timing is far less understood.

Circadian clocks are endogenous timekeepers that synchronize internal functions to daily rhythms in environmental demands [6]. At the molecular level, mammalian circadian clocks are composed of interlocked transcriptional-translational feedback loops. The transcription factors circadian locomotor output cycles kaput (CLOCK) and brain and muscle ARNT-like protein 1 (BMAL1 or ARNTL) drive expression of *Period* (*Per1-3*) and *Nuclear receptor subfamily 1 group D member1/2* (*Nr1d1/2* or *Reverbα/β*) genes during the day whose protein products are then during the night providing negative feedback on their own transcription [7,8]. Under alignment of the light–dark cycle with the feeding–fasting cycle, a master circadian pacemaker in the suprachiasmatic nucleus (SCN) of the hypothalamus synchronizes subordinate peripheral tissue clocks. When food intake is shifted into the inactive phase, however, it desynchronizes the circadian clock network and uncouples peripheral clocks from the SCN [9,10]. SCN clock-deficient mice gain body weight and show an impaired glucose metabolism in constant environmental conditions. However, this phenotype is rescued by reintroducing rhythmic feeding patterns, underlining the importance of circadian rhythms for the maintenance of metabolic homeostasis [11]. Rest-phase restricted feeding reduces energy expenditure and promotes body weight gain in mice compared to *ad libitum* food intake [12]. Nutrient derived signals feed back to the circadian clock network influencing peripheral tissue clocks [13,14].

Several studies in mice and humans point out that not only caloric consumption itself, but also the timing of food intake affects body weight regulation [2,15,16]. In rats, chocolate consumption in the beginning of the active phase is beneficial for circadian synchrony under shift-work and jet-lag conditions [17]. A human study in postmenopausal women revealed different effects of milk chocolate consumption in the morning or evening on appetite and energy expenditure [18]. We hypothesized that chronic rest phase snacking promotes body weight gain in male mice through a disruption of circadian clocks. Our study focuses on male mice as female mice are, e.g., protected from increased lipid accumulation under conditions of rhythm disruption through estrogen-effects on adipocyte clocks [19]. We fed mice a daily milk chocolate snack either in the early rest (daytime snack) or in the early active phase (nighttime snack) in addition to a normal chow diet and investigated body weight development and metabolic parameters. Our data show that chronic daytime snacking increases energy resorption rates and leads to body weight gain, a disruption of metabolic rhythms, and dampens intestinal clock gene expression. Interestingly, the effects of snack timing on body weight are preserved in wildtype mice in constant darkness (DD) and in clock deficient mice housed under a normal light–dark cycle but lost in clock mutant mice in DD. These data suggest that daytime snack effects require either a functional circadian clock or a rhythmic light–dark cycle.

## 2 Material and methods

### 2.1 Animals

**Figure 1:**
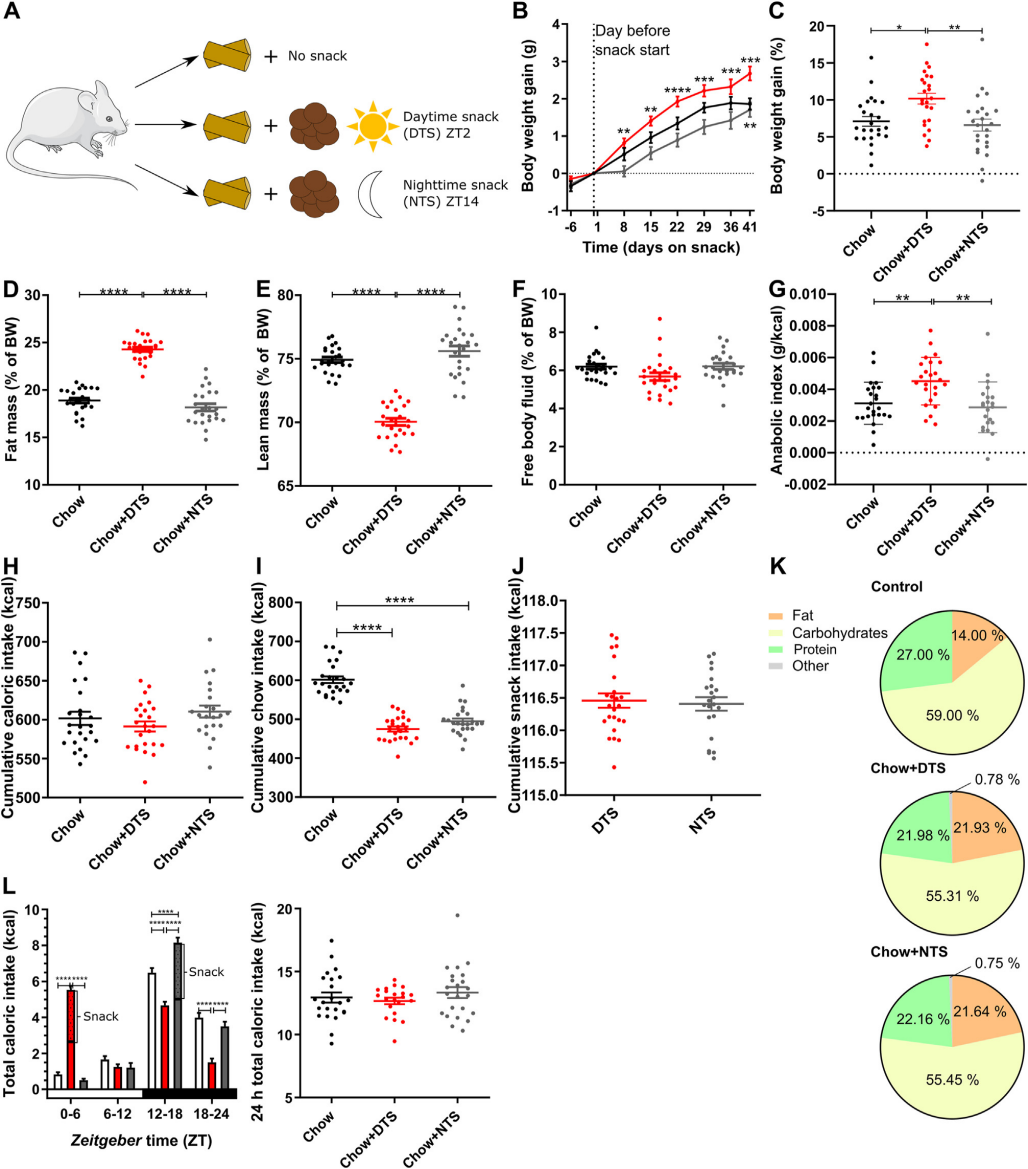
**Chronic daytime snacking promotes body weight gain without affecting cumulative caloric intake but by disrupting food intake rhythms.** A) Experimental setup. All mice received chow *ad libitum* and in addition no snack (control group), a daytime snack (DTS) at *zeitgeber* time (ZT) 2, or a nighttime snack (NTS) at ZT14. B) Body weight gain (g) normalized to the day before snacking was started. Bonferroni post-hoc test ∗∗p < 0.01, ∗∗∗p < 0.001, ∗∗∗∗p < 0.0001; ∗ above DTS vs. NTS, ∗ below chow vs. DTS; 2-way ANOVA: time and interaction p < 0.0001, group p < 0.001. C) Body weight gain (%) from the day before snacking was started until the end of the experiment. Bonferroni post-hoc test ∗ p < 0.05, ∗∗p < 0.01; 1-way ANOVA: p < 0.01. D) Fat mass, E) Lean mass, F) Free body fluid as percentage of body weight (BW). Bonferroni post-hoc test ∗∗∗∗p < 0.0001; 1-way ANOVA: D-E) p < 0.0001, F) p < 0.05. G) Anabolic index (total body weight gain (g)/cumulative caloric intake (kcal)), Bonferroni post-hoc test ∗∗p < 0.01, 1-way ANOVA: p < 0.001. H) Cumulative caloric intake; 1-way ANOVA: p > 0.05. I) Cumulative chow intake, Bonferroni post-hoc test ∗∗∗∗p < 0.0001, 1-way ANOVA: p < 0.0001. J) Cumulative snack intake, t-test: p > 0.05. K) Food composition (chow + snack) during 24 h. L) Food profile of total caloric intake after 1.5 weeks on snack and 24 h total caloric intake, Bonferroni post-hoc test ∗∗∗∗p < 0.0001; Mixed-effects analysis: time p < 0.0001, group p > 0.05, time x group p < 0.0001; 1-way ANOVA: p > 0.05. B–C, I) n = 24. D-H, J) chow and DTS n = 24, NTS n = 22. L) profile: chow and NTS n = 23–24, DTS n = 22–24, 24 h total caloric intake n = 21–23. Data are shown as mean ± SEM. Mouse image: smart.servier.com.

All animal experiments were performed in accordance with the German Law for Animal Welfare, reviewed by the state's ethical committee, and licensed by the Ministry of Energy, Agriculture, Environment, Nature, and Digitalization (MELUND) of the State of Schleswig–Holstein, Germany. Adult male C57BL/6J wildtype mice were purchased from Janvier Labs (Le Genest-Saint-Isle, France), adult male homozygous *Per1/2* double mutant mice (*B6.CgPer1*^*tmBrd*^*Tyr*^*c-Brd*^*/J* and *B6.CgPer2*^*tmBrd*^*Tyr*^*c-Brd*^*/J*) on a C57BL/6J background were raised in the breeding facility of the University of Lübeck, Germany. *Per1/2* double mutants are arrhythmic in DD but show rhythmic activity patterns when kept in a 12h:12h light–dark cycle (LD) [20]. We used *Per1/2* double mutant mice as clock-deficient mouse model as *Bmal1*-KO mice have an age-related phenotype and already stop gaining body weight with 16 weeks of age [21]. Mice were single-housed in LD or DD under standard laboratory conditions with *ad libitum* access to normal chow and tap water. Mice were divided into three age- and weight-matched experimental groups. While two groups of mice received a daily chocolate snack either in the early rest phase at *zeitgeber* time (ZT; ZT0 = light onset) 2 (daytime snack (DTS)) or in the early active phase at ZT14 (nighttime snack (NTS)), the third group of mice (chow control group) did not receive any snack (Figure 1A). In the DD experiment, the endogenous period of the wildtype mice was determined over two weeks from infrared locomotor activity data in ClockLab (Actimetrics, Wilmette, USA). Afterwards, mice were entrained for one week in LD before snacking in DD was started. The mean endogenous period of the wildtype mice was used to determine the correct circadian time (CT; CT0 = beginning of subjective day, CT12 = beginning of subjective night) for daytime (CT2) or nighttime (CT14) snacking, respectively, in wildtype as well as *Per1/2* double mutant mice. Measurements in the experimental groups were performed simultaneously to avoid batch effects. A timeline figure (Suppl. Figure 1) indicates which of the following measurements were performed at which time during the experimental period.

### 2.2 Body weight, anabolic index, and body composition

Body weight of mice was determined weekly at ZT8. To calculate body weight gain during snacking, body weight data were normalized to the day before daily snacking was started. For comparing body weight gain after two weeks in wildtypes in LD, body weight data from the long-term snacking experiment were used. Therefore, the number of mice was much larger in these cohorts (wildtypes LD n = 24, *Per1/2* double mutants LD n = 6, wildtypes and *Per1/2* double mutants DD n = 8). To control for potentially different sizes and thereby differences in body weight gain and cumulative food intake of the mice, the anabolic index was calculated by dividing body weight gain over the whole experiment by the cumulative caloric intake (control and DTS groups: n = 24, NTS group: n = 22). Body composition was measured at ZT8 close to the end of the experiment (end of week 5, n = 24) using a Bruker Minispec LF110 NMR (Bruker, Billerica, USA). Data were calculated in percent of total body weight, whereby the total body weight determined by the NMR consisted of lean mass, fat mass, and free body fluid.

### 2.3 Food intake and composition

Chow (breeding diet #1314, Altromin, Lage, Germany; 14% fat, 27% protein, 59% carbohydrates, metabolized energy: ∼3,339 kcal/kg) intake during the snacking experiment contained chow intake data from ZT0 seven days prior to snack start until ZT8 after 5 weeks and 5 days on daily snacking (n = 24 per group). During the experiment, mice received a daily chocolate snack (0.5–0.6 g RUF milk chocolate drops, RUF, Quakenbrück, Germany; per 100 g: 2,099/2,276 kJ = 503/545 kcal, fat 27.1/32 g from which 16.7/19 g saturated fatty acids, carbohydrates 54/57 g from which 47.6/56 g sugar, protein 6.5/6.8 g, salt 0/0.2 g). These data were summarized for the cumulative snack intake and added to the total chow intake data to calculate the cumulative caloric intake (control and DTS groups: n = 24, NTS group: n = 22). The average cumulative caloric intake per 24 h was calculated by dividing the total intake during the two experimental weeks by the number of days. The manufacturer's indications were used to calculate food composition. Food was weighed every 6 h to calculate daily caloric intake rhythms (food profile 1.5 weeks on snack: n = 22–24, 24 h caloric intake on 1.5 weeks food profile day n = 21–23). In the profiles before and at 1.5 weeks on snacking, 10 data points each were excluded due to technical issues with the scales or handling issues with small food crumbs in the dark. In the 24 h intake calculation 5 data points were excluded. Therefore, the above indicated number of mice within the groups is varying.

### 2.4 Indirect calorimetry

Respiratory exchange ratio (RER) was determined from oxygen consumption and carbon dioxide production rates using an open-circuit indirect calorimetry system (TSE Systems, Berlin, Germany). The measurement was performed during the first week of the snacking regimen (n = 8). To avoid frequent opening of the TSE system during snack provision, DTS and NTS were measured in two batches but always simultaneous to chow control mice (1. Chow control and NTS mice, 2. Chow control and DTS mice). During the first experimental week, mice were comparable in body weight and ANCOVA analysis using body weight as co-variant did not reveal a significant difference in energy expenditure between the groups [22]. Energy expenditure was determined as follows using caloric equivalents according to Heldmaier [23]: $energy\;expenditure\;\left[\frac{kJ}{h}\right]=\left(4.44+1.43\times RER\right)\times VO_{2}\left[\frac{mL\;O_{2}}{h}\right]\times 0.0036$.

### 2.5 Locomotor activity and core body temperature

Activity represented in actograms was recorded in ClockLab (Actimetrics) using infrared detectors. For additional analyses, locomotor activity as well as core body temperature were measured using G2 E-mitters (Starr Life Sciences, Oakmont, USA) implanted into the intraperitoneal space. Mice anesthetized with isoflurane (4% in air) were injected with 4 mg/kg Carprofen (Rimadyl, Zoetis, Parsippany, USA). Eyes were prevented from dehydration using Bepanthen (Bayer, Leverkusen, Germany). To implant the sterilized E-mitter, the abdomen was shaved and disinfected. The skin and the muscular layer above the abdominal cavity were opened and closed separately. After one week of recovery, temperature and activity were recorded in 1-min intervals utilizing ER4000 receivers (Starr Life Sciences) and the Vital View software (version 5, Starr Life Sciences). Locomotor activity and core body temperature were recorded before and during snacking. Due to technical issues with the recordings, the number of mice within the different groups varied (n = 4–8). To investigate locomotor activity and core body temperature development around the snacking time over the course of the experiment, total activity as well as the mean body temperature were calculated on three baseline and 14 experimental days from ZT0-6 and ZT12-18 each (n = 4–10). Daily locomotor activity profiles and total activity of wildtype and *Per1/2* double mutant mice in LD and DD were compared using data recorded with infrared detectors. Activity was averaged over three days.

### 2.6 Infrared thermography

Interscapular brown adipose tissue (iBAT), tail, and inner-ear temperature were measured in the third week of snacking at ZT3 and 15 using an infrared camera (T335, thermal sensitivity of <0.05 °C, accuracy of 2% max 2 °C, FLIR, Wilsonville, USA). During the measurement, mice (n = 8–9) moved freely on the grid of their home cage. Reproducibility of iBAT images was enhanced by using vaseline (Balea, dm, Karlsruhe, Germany) as described before [24]. Inner-ear, tail, and iBAT temperatures were determined from the maximum temperature of three images and evaluated in FLIR Tools Version 5.13.18031.2002 (FLIR) and FLIR QuickReport Version 1.2 (FLIR). The inner-ear temperature was used as an approximation for core body temperature for normalization.

### 2.7 Tissue and serum/plasma collection

Serum, duodenum, and jejunum samples were isolated from mice sacrificed by cervical dislocation after six weeks of snacking. Intestine samples were washed in 1× DPBS (Gibco, Life Technologies Europe B.V., Beiswijk, The Netherlands) and transferred into RNAlater (Invitrogen, ThermoFisher Scientific, Waltham, USA). Samples were incubated with RNAlater for 9–12 h at 4 °C before they were stored at −20 °C. Blood was allowed to clot for 20 min at room temperature and subsequently centrifuged for 30 min at 664 rcf and 4 °C. Blood for plasma collection after three weeks of snacking was sampled in EDTA-coated tubes (K3E microvette 500, Sarstedt, Nümbrecht, Germany) obtained by heart puncture. Samples were centrifuged for 30 min at 620 rcf and 4 °C. Serum and plasma samples were stored at −80 °C.

### 2.8 Serum glucose, insulin, triacylglyceride (TAG), free glycerol levels, total cholesterol and cholesterol from lipoprotein fractions

A glucometer (ACCU-CHECK, Aviva, Roche, Mannheim, Germany) was used to determine serum glucose concentrations (mg/dL, n = 5–6). Glucose levels were in a comparable range to other circadian profiles of random-fed chow mice [[25], [26], [27], [28]], however we cannot completely exclude that mice were not stressed by the handling. Triacylglyceride (TAG, n = 4–6) and insulin (n = 4–6) levels were measured in duplicates with a triglyceride colorimetric assay kit (item no. 10010303, Cayman Chemical, Ann Arbor, USA) and a mouse insulin ELISA (article no. 10-1247-01, 5 μL protocol, Mercodia, Uppsala, Sweden) following the manufacturers' instructions. Three samples were measured in singlets due to material limitations. Total cholesterol levels (n = 3–6) were determined in duplicates using a total cholesterol colorimetric assay kit (article no. STA-384, Cell Biolabs, San Diego, USA) following the manufacturer's instructions. The assay also measured cholesterol from cholesterylesters. To measure free glycerol levels (n = 3–6) in singlets a free glycerol assay kit (II, colorimetric, article no. ab1558899, Abcam, Cambridge, UK) was used according to the manual using 2 μL of serum. Two outliers in TAG and free glycerol levels and one outlier in insulin and cholesterol levels were detected by Grubbs outlier test and excluded. To determine TAG, cholesterol and free glycerol levels, the blank absorbance value was subtracted from each duplicate mean and the concentration calculated using the standard curve. A four-parameter logistic curve was fitted through the insulin standards to calculate insulin levels which were converted as follows: 1 μg ≙ 174 pmol and 6 pmol/L ≙ 1 μIU/mL. Cholesterol from lipoprotein fractions and total cholesterol was measured in singlets with a colorimetric assay kit (no. E2HL-100, Bioassay systems, Hayward, USA) according to the manufacturer's instructions. Due to material limitations serum samples of four time points over the 24h day were pooled (n = 3–5). Samples were diluted 1:2 in assay buffer. Total cholesterol and cholesterol from the lipoprotein fractions were calculated according to the kit's manual by subtracting the blank absorbance value.

### 2.9 Lipidomics

An untargeted lipidomic screen was carried out as described previously [29] and conducted on plasma samples (ZT3 and 15) of mice after three weeks on daily snacking using a Dionex Ultimate 3000 LC system (ThermoFischer Scientific, Bremen, Germany) combined with an Orbitrab mass spectrometer (QExactive, ThermoFisher Scientific). For lipid extraction 50 μL plasma were mixed for 30 s with 1 mL 4 °C methanol:tert-butylmethylether:chloroform (1.33:1:1, v/v/v; methanol hypergrade for LC-MS (LiChrosolv, Supelco, Bellefonte, USA), tert-butylmethylether and chloroform (chromasolv plus for HPLC ≥99.9%, Honeywell, Riedel-de Haën, Charlotte, USA)) containing butylated hydroxytoluene (100 mg/L; Sigma-Aldrich, St. Louis, USA) and SPLASH Lipidomix Mass Spec Standard (2.5 μL/mL; SKU-330707-1EA (Avanti Polar Lipids, Birmingham, USA)). Samples were further incubated for 30 min at 950 rpm (ThermoMixer C, Eppendorf, Hamburg, Germany) and 25 °C, mixed for 30 s, followed by a 10-min centrifugation step at 2,000 rcf and 20 °C. Supernatants were evaporated for 2.5 h in a SpeedVac (refrigerated Vapor Trap Savant RVT5105, SpeedVac concentrator Savant SPD111V, Vacuum pump VLP120; Thermo Fisher Scientific, Waltham, USA) at room temperature and resuspended in 50 μL methanol:isopropanol (1:1). Samples were mixed for 10 s and centrifuged at 20,817 rcf and 20 °C for 10 min. Due to limited plasma, only 40 μL were used for two samples and the volumes in the protocol reduced accordingly. Lipids (injection volume: 5 μL) were separated on an Accucore C30 (150 mm/2.1 mm, 2.6 μm; Thermo Fisher Scientific) column. The mobile phase consisted of eluent A – acetonitrile:H_2_O (6:4; LC-MS grade water (LiChrosolv)) with 10 mM NH_4_CH_3_COO (0.7708 g/L; ammonium acetate for mass spectrometry (Sigma-Aldrich)) – and eluent B – isopropanol:acetonitrile (9:1, v/v; 2-propanol chromasolv LC-MS ≥ 99.9% (Honeywell)) with 10 mM NH_4_CH_3_COO. Formic acid (0.1%, Biosolve Chimie, Dieuze, France) was added to both eluents. A flow rate of 0.26 mL/min and the following gradient were used: increasing gradient of B 30% B at 0 min, 43% B at 2 min, 55% B at 2.1 min, 65% B at 12 min, 85% B at 18 min, 100% B at 20 min. Afterwards the column was washed for 15 min with 100% B and re-equilibrated for 3 min with the starting mobile phase. Ionization and data-dependent MS/MS acquisition were performed as described previously [29,30]. Lipids were identified using an in-house library. The determined area under the peak was normalized to the internal standard. Pooled plasma samples at five different concentrations were processed as quality controls. 50 μL of LC-MS grade water (LiChrosolv, Supleco) was extracted with the samples to exclude random signals.

### 2.10 Bomb calorimetry

Feces were collected in 6-hour intervals from ZT0-6 and 12–18 (n = 6–8) after five weeks on snacking. Five data points could not be measured as either no or insufficient amounts of feces were provided. Before measurement food samples and feces were dehydrated until weight stability. The energy intake was calculated by multiplying the consumed food (g) with the energy content in the respective food samples (J/g). Food samples were either chow or representative food mixtures (chow + chocolate) specifically for either the DTS group at ZT0-6 or the NTS group at ZT12-18. The energy of the feces (excreted energy) was measured using a C200 Oxygen Bomb Calorimeter (IKA, Staufen, Germany). The assimilated energy was calculated by subtracting the excreted energy from the energy intake.

### 2.11 RNA isolation and quantitative real-time PCR (qPCR)

Trizol- (Ambion, Life Technologies, Austin, USA) chloroform (≥99.5%, Honeywell, Charlotte, USA) extraction was used to isolate total RNA from tissue homogenates (Omni Bead Ruptor 24, Omni International Kennesaw, USA). cDNA was generated by reverse transcription of the RNA using the high-capacity cDNA reverse transcription kit (Applied Biosystems, Waltham, USA) following the manufacturer's protocol. Gene expression was determined by qPCR using the Go Taq qPCR master mix kit (Promega, Madison, USA) and a CFX-96 thermocycler (Bio-Rad, Hercules, USA). Relative mRNA expression was obtained by analyzing gene expression with the ΔΔC_t_ method using *Eef1α* as housekeeping gene. Data were normalized to the mean ratio of the chow control group (n = 4–6). Primer sequences were: *Bmal1* forward 5′-CCTAATTCTCAGGGCAGCAGAT-3′, *Bmal1* reverse 5′-TCCAGTCTTGGCATCAATGAGT-3′, *CD36* forward 5′-TGAATGGTTGAGACCCCGTG-3′, *CD36* reverse 5′-TAGAACAGCTTGCTTGCCCA-3′, *Dbp* forward 5′-AATGACCTTTGAACCTGATCCCGCT-3′, *Dbp* reverse 5′-GCTCCAGTACTTCTCATCCTTCTGT-3′, *Dgat1* forward 5′-TCCGTCCAGGGTGGTAGTG-3′, *Dgat1* reverse 5′-TGAACAAAGAATCTTGCAGACGA-3′, *Dgat2* forward 5′-TTCCTGGCATAAGGCCCTATT-3′, *Dgat2* reverse 5′-ACTCTATGGTGTCTCGGTTGAC-3′, *Eef1α* forward 5′-TGCCCCAGGACACAGAGACTTCA-3′, *Eef1α* reverse 5′- AATTCACCAACACCAGCAGCAA-3′, *Nr1d1* forward 5′-AGCTCAACTCCCTGGCACTTAC-3′, *Nr1d1* reverse 5′-CTTCTCGGAATGCATGTTGTTC-3′, *Slc2a2* forward 5′-TCAGAAGACAAGATCACCGGA-3′, *Slc2a2* reverse 5′-GCTGGTGTGACTGTAAGTGGG-3′, *Slc5a1* forward 5′-TCTGTAGTGGCAAGGGGAAG-3′, *Slc5a1* reverse 5′-ACAGGGCTTCTGTGTCTTGG-3’.

### 2.12 Statistical analysis

All data are expressed as group mean ± SEM. GraphPad Prism 8 (GraphPad, San Diego, USA) was used for statistical comparisons considering p-values <0.05 as significant. T-tests were performed to compare two groups. One-way analysis of variance (ANOVA) was used to compare three groups, 2-way ANOVA/mixed effects analysis to compare data between groups and different ZTs. If applicable, repeated measurement statistics were used. In all cases Bonferroni post-hoc tests were used. Outliers based on Grubbs outlier test were excluded in qPCR and serum parameter data. Rhythmicity was assessed by Circwave, version 1.4 (p-value cut-off 0.05) [31]. Heat maps were created using metaboanalyst.ca, version 5.0, with Ward clustering algorithm and Euclidean distance measurement.

## 3 Results

### 3.1 Daytime snacking promotes weight gain and increased fat mass in male mice independent of caloric intake

To investigate the effect of timed snacking on metabolic homeostasis, we performed a chronic chocolate snack experiment. In addition to chow *ad libitum* mice received a daily snack either during their daily rest phase at ZT2 (daytime snack, DTS) or their active phase at ZT14 (nighttime snack, NTS), respectively (Figure 1A). An additional control group (chow) received no snack. DTS mice continuously gained body weight over the course of the experiment reaching a mean body weight gain of 2.7 g (+10.2%) at the end of the experiment (Figure 1B,C). NTS and control mice increased body weight to a lesser extent (1.7 g (6.6%) and 1.9 g (7.1%), respectively), whereby NTS mice were always comparable to the control group (Figure 1B,C). After six weeks of snacking, DTS mice gained significantly more body weight compared to the control and NTS groups (Figure 1C). In line with this, at the end of the experiment DTS mice showed increased fat and decreased lean mass compared to the chow and NTS groups which showed very similar levels of fat and lean mass (Figure 1D–E). Free body fluid was comparable in all three groups (Figure 1F). We additionally calculated the anabolic index which was – in line with the previous results – increased in DTS and unaltered between the control group and NTS mice indicative of increased energy uptake and storage in DTS mice (Figure 1G). In contrast, cumulative caloric ingestion was comparable in all three groups (Figure 1H). DTS and NTS mice reduced their chow intake by roughly the number of calories consumed through the snack (Figure 1I–J). Notably, both snacking groups consumed similar amounts of chocolate (Figure 1J). We next analyzed whether food composition was altered between the experimental groups throughout a 24-hour day. Both snacking groups consumed slightly more fat and less carbohydrates and proteins compared to the chow control group (Figure 1K). Overall, food composition was comparable between DTS and NTS mice (Figure 1K) but was prominently altered during the snacking time itself suggesting a shift in the daily profile of nutrient composition (Suppl. Figure A.2A). We therefore investigated daily food intake rhythms in these mice. Already in the second week of the experiment nighttime snacking augmented the natural calorie intake rhythm whereas daytime snacking let to increased intake during the first half of the rest phase and reduced food intake during the dark phase (Figure 1L). Again, 24-hour total caloric intake was comparable between all groups (Figure 1L). Notably, chronic timed daytime snacking also influenced chow-only intake rhythms with a higher chow consumption in the early rest phase (Suppl. Figure A.2B). Together, these data indicate that chronic daytime snacking promotes body weight gain associated with altered diurnal food intake and diet composition rhythms.

### 3.2 Daytime snacking reduces active-phase energy expenditure

**Figure 2:**
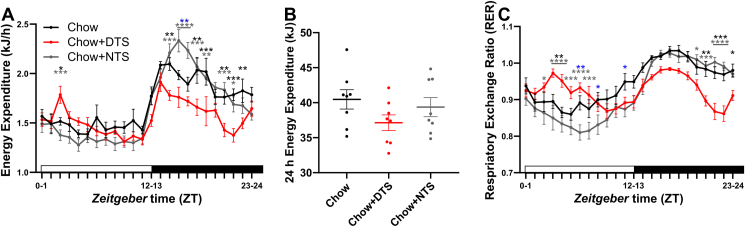
**Daytime snacking alters daily metabolism.** A) Energy expenditure at day 6 on snacking. B) Total daily (24 h) energy expenditure. 1-way ANOVA: p > 0.05. C) Respiratory exchange ratio (RER) at day 6 on snacking; A,C) Bonferroni post-hoc test ∗ p < 0.05, ∗∗p < 0.01, ∗∗∗p < 0.001, ∗∗∗∗p < 0.0001, ∗ chow vs. DTS,  chow vs. NTS,  DTS vs. NTS; 2-way ANOVA: time, interaction p < 0.0001, group p > 0.05. A-C) Data are shown as mean ± SEM; n = 8. DTS: daytime snack, NTS: nighttime snack.

We next sought to determine how timed snacking affects diurnal energy turnover. Energy expenditure and RER were determined on day 6 of the experiment in an indirect calorimetry system (Figure 2). During the first week of snacking, the three groups were comparable in body weight. When we plotted energy expenditure vs. body weight (Suppl. Figure A.3D-E), ANCOVA analysis using body weight as co-factor revealed no significant difference in energy expenditure between the groups [22]. Nighttime snacking stabilized the diurnal rhythm of energy metabolism. In NTS mice, energy expenditure was even higher during snacking resulting in an exaggerated diurnal rhythm compared to chow mice (Figure 2A). Daytime snacking had the opposite effect increasing energy expenditure during the rest phase (*i.e.*, the snacking time) and reducing energy expenditure throughout the active phase (Figure 2A). Over the 24-hour day, energy expenditure was slightly reduced in the DTS group, but this effect was not significant (Figure 2B). Notably, we already found similar trends on the second day of snacking (Suppl. Figure A.3). In line with altered food composition profiles throughout the day and specifically around snacking (Figure 1K and Suppl. Figure A.2A), we observed snack-induced changes in the daily RER rhythm. While nighttime snacking reduced RER throughout the rest phase, daytime snacking disrupted daily metabolic profiles by increasing the RER during and after the snacking time (Figure 2C). Additionally, RER was reduced throughout the active phase upon chronic daytime snacking (Figure 2C). In line with the increase in body weight observed in the DTS group, our data show reduced energy expenditure throughout the active phase after daytime snacking in these mice indicating a disruption of daily energy metabolism.

### 3.3 Daytime snacking affects blood lipid profiles

**Figure 3:**
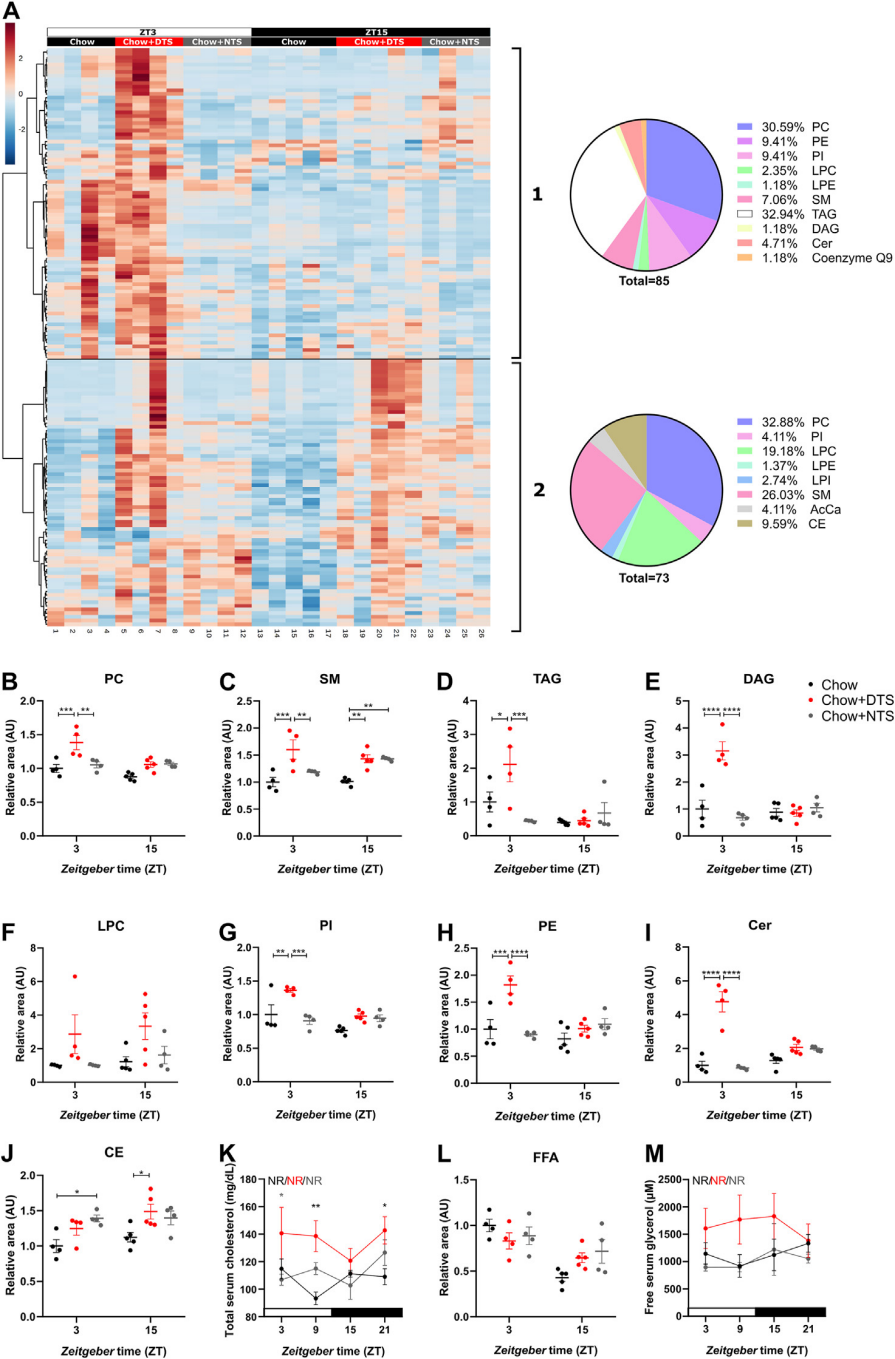
**Daytime snacking increases plasma lipids at ZT3.** A) Heat map of lipids detected in plasma after three weeks on snacking at *zeitgeber* time (ZT) 3 and 15 during the lipidomics screen. Lipids are listed in order of appearance in Supplemental Table B. Pie charts describe the amount of lipids of the respective class in cluster 1 and 2. B-J,L) Lipids summarized in their respective lipid classes (cluster 1 and 2 together): B) Phosphatidylcholines (PC), C) Sphingomyelins (SM), D) Triacylglycerides (TAG), E) Diacylglyceride (DAG), F) Lysophosphatidylcholines (LPC), G) Phosphytidylinositols (PI), H) Phosphatidylethanolamines (PE), I) Ceramides (Cer), J) Cholesterylesters (CE), L) Free fatty acids. Further lipid classes are presented in Supplementary Figure A.4. K) Total serum cholesterol and M) free serum glycerol after six weeks on snacking. B-M) Bonferroni post-hoc test ∗ p < 0.05, ∗∗p < 0.01, ∗∗∗p < 0.001, ∗∗∗∗p < 0.0001, ∗ chow vs. DTS,  DTS vs. NTS; 2-way ANOVA: B) time p < 0.01, group p < 0.001, interaction p < 0.05; C) time, interaction p > 0.05, group p < 0.0001; D) time, interaction p < 0.01, group p < 0.05; E) time p < 0.001, group, interaction p < 0.0001; F) time, interaction p > 0.05, group p < 0.01; G) time p < 0.01, group p < 0.001, interaction p < 0.05; H) time p < 0.05, group p < 0.001, interaction p < 0.01; I) time p > 0.05, group, interaction p < 0.0001; J) time, interaction p > 0.05, group p < 0.01; K) time, interaction p > 0.05, group p < 0.001; L) time p < 0.001, group p > 0.05, interaction p < 0.05; M) time, interaction p > 0.05, group p < 0.01. Data are shown as mean ± SEM; B-J,L) n = 4–5; K-M) n = 3–6. AcCA: Acylcarnitines, AU: arbitrary units, DTS: daytime snack, LPE: Lysophosphatidylethanolamines, LPI: Lysophosphatidylinositols, NTS: nighttime snack, NR: not rhythmic.

The higher anabolic index (Figure 1G) observed in DTS mice together with slightly, but not significantly decreased energy expenditure (Figure 2B) and altered fuel utilization (Figure 2C) prompted us to test how timed snacking affects nutrient uptake into the blood. As a first step we performed a plasma lipidomics screen at ZT3 and ZT15 with or without prior snacking after three weeks on the snacking schedule (Figure 3). When we plotted all detected lipids in a heat map (listed in Suppl. Table B in order of appearance in the heat map), we found two main clusters. In the first cluster plasma lipids were increased at ZT3 – 1 h after the DTS – in the DTS group while lipid levels were unaltered in the control and NTS groups (Figure 3A). Of note, we observed a slight upregulation in some plasma lipids at ZT3 in the chow control group, however, this was only seen in one mouse (Figure 3A). The first cluster mainly contained triacylglycerides (TAG), phosphatidylcholines (PC), phosphatidylethanolamines (PE), and phosphatidylinositols (PI) (Figure 3A). In the second cluster we observed a general snack-induced increase in plasma lipids in both snacking groups at both time points whereby the overall increase in the DTS group was still stronger compared to the NTS group (Figure 3A). This second cluster was mainly composed out of PC, sphingomyelins (SM), and lysophosphatidylcholines (LPC) (Figure 3A).

We then focused on the largest lipid chemical classes. We normalized each compound to its mean in the ZT3 chow control group and grouped the lipids according to their classes. Notably, some lipid classes such as, e.g., SM, PC, and LPC were represented in both clusters (Figure 3A) and we therefore combined lipids from cluster 1 and 2 in the grouped analysis. We found a significant increase of PC at ZT3 in the DTS group compared to the chow and NTS groups (Figure 3B). For SM we observed the same, but in addition we found a snack-induced increase at ZT15 (Figure 3C). Daytime snacking also increased TAG and Diacylglyceride (DAG) levels at ZT3 (Figure 3D–E) compared to the control and NTS groups. LPC levels were not significantly altered but represented the general elevated levels in the DTS group at both time points (Figure 3F). PI, PE and ceramide (Cer) levels were increased at ZT3 in the DTS group (Figure 3G–I). Cholesterylesters were significantly increased in the NTS group at ZT3 and in the DTS group at ZT15 compared to the control group (Figure 3J). We therefore investigated total serum cholesterol levels after six weeks of snacking. Total cholesterol was increased in the DTS group suggesting dyslipidemia (Figure 3K). In line, cholesterol from HDL or LDL/VLDL lipoprotein fractions were increased in the DTS compared to the control and NTS group (Suppl. Figure A.4E). Free fatty acids were not altered (Figure 3L). Additionally, we analyzed free serum glycerol levels after six weeks of snacking. Overall, free glycerol was elevated in the DTS group but did not reach significance in post-hoc comparisons (Figure 3M). We also observed changes in additional lipid classes (Suppl. Figure A.4). These changes were mostly restricted to a significant increase in the DTS compared to the chow group at ZT15 for acylcarnitines (AcCA) and lyosphosphatidylethanolamines (LPE) (Suppl. Figure A.4). Coenzyme Q9 was elevated at ZT3 compared to the chow and NTS groups. Lysophosphatidylinositols (LPI) were not altered (Suppl. Figure A.4). Taken together, these results suggest that not snacking in general but specifically daytime snacking strongly affects plasma lipid levels.

### 3.4 Daytime snacking alters daily locomotor activity as well as core body temperature rhythms and increases activity and temperature after the snack

**Figure 4:**
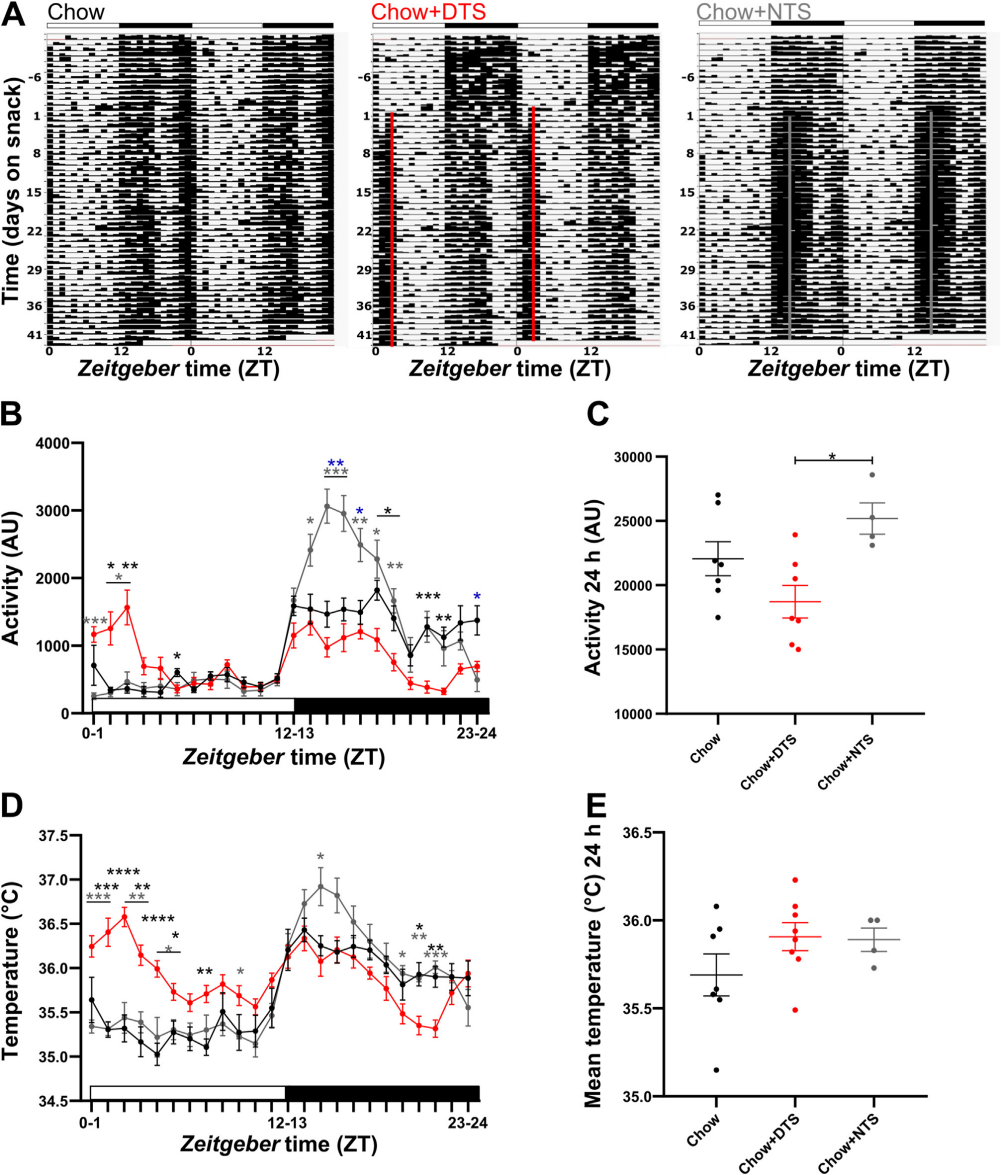
**Daytime snacking alters locomotor activity and core body temperature rhythms.** A) Representative actograms for chow, daytime snack (DTS) and nighttime snack (NTS) mice showing activity measured by infrared sensors. B-E) Activity (B,C) and temperature (D,E) during day 6–8 on snacking in B,D) 1 h and C,E) 24 h intervals measured by E-mitter sensors. B) Bonferroni post-hoc test ∗ p < 0.05, ∗∗p < 0.01, ∗∗∗p < 0.001, ∗ chow vs. DTS,  chow vs. NTS,  DTS vs. NTS; Mixed-effects analysis: time, time x group p < 0.0001, group p < 0.01. C) Bonferroni post-hoc test ∗ p < 0.05; 1-way ANOVA: p < 0.05. D) Bonferroni post-hoc test ∗ p < 0.05, ∗∗p < 0.01, ∗∗∗p < 0.001, ∗∗∗∗p < 0.0001, ∗ chow vs. DTS, ∗ chow vs. NTS, ∗ DTS vs. NTS; Mixed-effects analysis: time, time x group p < 0.0001, group p > 0.05. E) 1-way ANOVA: p > 0.05. B-E) Data are shown as mean ± SEM; n = 4–8 per ZT and group. AU: arbitrary units.

Due to the disturbed feeding pattern as well as changed metabolism after daytime snacking, we next investigated whether daily locomotor activity and core body temperature rhythms were disturbed. Snack timing differently affected locomotor activity rhythms from the first days of snacking onwards (Figure 4A–C). Representative actograms of mice receiving a daily snack indicate reduced activity of DTS mice in the second half of the active phase together with food anticipatory activity (FAA) before the snacking time compared to the control group (Figure 4A). At the same time, NTS mice had a more condensed activity pattern in the first half of the active phase indicating a stabilization of the diurnal activity rhythm due to the snack (Figure 4A). Daytime snacking induced FAA as well as activity during the snacking itself and let to reduced activity throughout the night (Figure 4B). Nighttime snacking strongly increased activity during the first half of the active phase (Figure 4B). 24-hour activity was slightly reduced in the DTS and slightly increased in the NTS group compared to the chow group without reaching significance; however, we observed a significant difference between DTS and NTS mice (Figure 4C). In line with changes in locomotor activity, the time of snacking affected core body temperature rhythms (Figure 4D–E). Already after a few days of snacking, DTS mice showed increased temperature before the snack as well as increased postprandial thermogenesis after the snack which resulted in a flattened rhythm with two daily peaks (Figure 4D). Additionally, thermogenesis was reduced at the end of the active phase (Figure 4D). Nighttime snacking increased postprandial thermogenesis as a direct response to the snack (Figure 4D). The mean daily temperature was mildly increased in both snacking groups without reaching significance (Figure 4E).

To investigate whether increased iBAT thermogenesis or altered heat dissipation via the tail are involved in the changes in core body temperature upon snacking, we analyzed infrared pictures of the iBAT, tail, and inner-ear (as a readout of core body temperature, Suppl. Figure A.5). Compared to chow mice, DTS and NTS mice showed slight alterations in the inner-ear and iBAT surface temperature that resulted in a significantly higher inner-ear and iBAT temperature in DTS compared to NTS mice (Suppl. Figure A.5C,E). However, normalization of the iBAT surface temperature to inner-ear temperature abolished these differences, showing that BAT is not fully responsible for the observed increase in core body temperature of DTS mice during the rest phase (ZT3, Suppl. Figure A.5G). Furthermore, and in line with a centrally induced increase in body temperature (pyrexia), tail surface temperature of DTS mice was not changed (Suppl. Figure A.5D). Interestingly, NTS mice displayed an increased tail surface temperature compared to DTS mice when normalized to inner-ear temperature which shows that they used heat dissipation via the tail to actively lower their body temperature during the rest phase (ZT3, Suppl. Figure A.5F). Taken together, these results indicate that snacking induces changes in the central regulation of the body temperature set-point during the rest phase of mice, while nighttime body temperature is not affected (ZT15, Suppl. Figure A.5).

**Figure 5:**
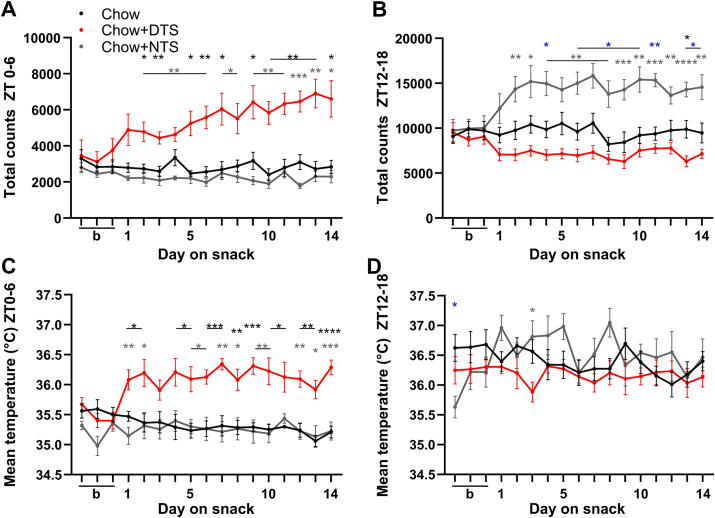
**Locomotor activity and temperature increase after daytime snacking.** A, B) Locomotor activity and C, D) mean core body temperature from A,C) *zeitgeber* time (ZT) 0–6 and B,D) ZT12-18 during three baseline (b) and 14 experimental days. Bonferroni post-hoc test ∗ p < 0.05, ∗∗p < 0.01, ∗∗∗p < 0.001, ∗∗∗∗p < 0.0001, ∗ chow vs. DTS,  chow vs. NTS,  DTS vs. NTS; Mixed-effects analysis: A,B) time, group p < 0.001, time x group p < 0.0001; C) group, time x group p < 0.0001, time p > 0.05; D) time x group p < 0.01, group, time p > 0.05. A-D) Data are shown as mean ± SEM; n = 4–10 per day and group. DTS: daytime snack, NTS: nighttime snack.

Due to the observed changes in locomotor activity and core body temperature we next investigated how the increases in response to the snack developed during the first two weeks of the experiment (Figure 5). While locomotor activity and mean core body temperature at ZT0-6 were comparable in the chow and NTS groups, daytime snacking led to a direct increase in both, locomotor activity and body temperature (Figure 5A,C). Daytime snacking induced a continuous increase in locomotor activity throughout the first half of the inactive phase over the whole two weeks of the experiment (Figure 5A), whereas the mean core body temperature during this time reached its plateau already on the first day (Figure 5C). Total activity from ZT12-18 was mostly comparable in the chow and DTS groups, whereas nighttime snacking increased activity during the first three days and was then reaching a plateau (Figure 5B). The mean core body temperature from ZT12-18, on the other hand, was comparable between all three groups. Thus, nighttime snacking did not induce temperature increase (Figure 5D). Overall, daytime snacking increased locomotor activity and core body temperature, whereas nighttime snacking only led to an increase in locomotor activity.

Taken together, these data show disruptions in locomotor activity as well as disturbed core body temperature rhythms from the first days of daytime snacking onwards. These reductions in activity and temperature during the active phase and contrastingly increases in activity and temperature in the beginning of the normal rest phase in the daytime snack group could further contribute to the elevated body weight gain in DTS mice.

### 3.5 Daytime snacking is affecting daily energy equivalents in glucose and TAG and is dampening intestinal clock gene expression

**Figure 6:**
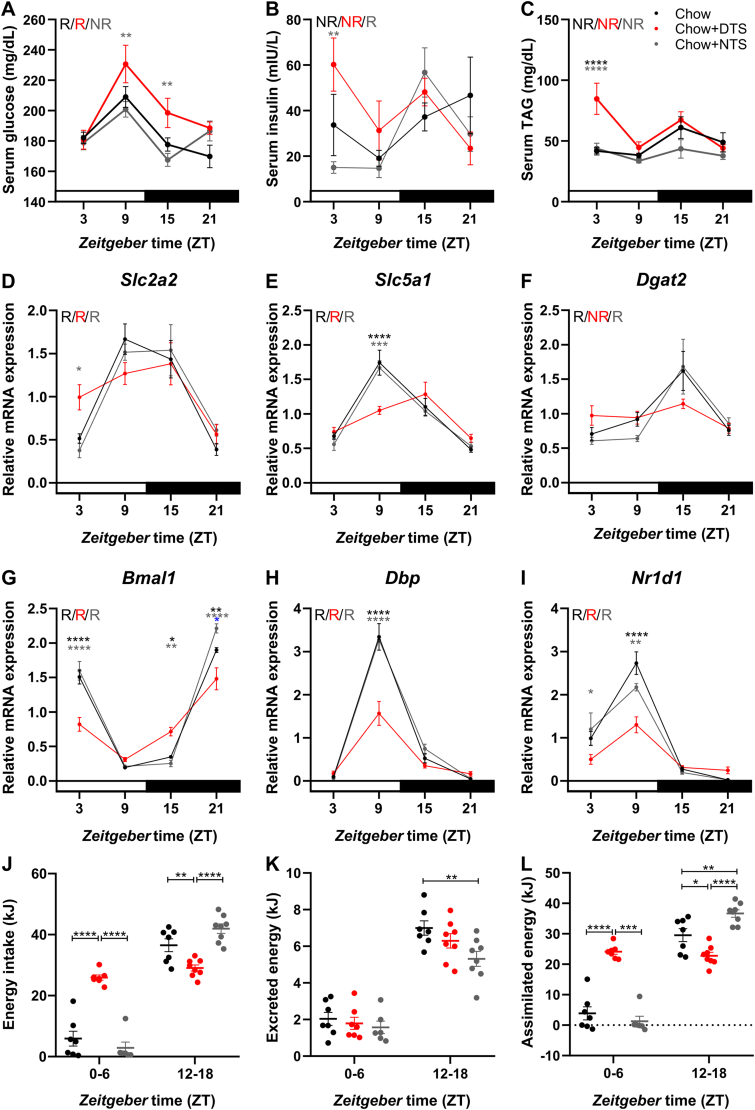
**Daytime snacking alters energy equivalents, expression of genes involved in nutrient utilization as well as clock genes in the jejunum and increases total assimilated energy.** Serum A) glucose, B) insulin, and C) triacylglyceride (TAG) levels after six weeks on snacking. Expression of glucose uptake transporter genes D) *Slc2a2*, E) *Slc5a1*, F) *Dgat2* and clock genes G) *Bmal1*, H) *Dbp*, I) *Nr1d1* in the jejunum after six weeks on snacking. J) Energy intake, K) excreted and L) assimilated energy after five weeks on snacking. Bonferroni post-hoc test ∗ p < 0.05, ∗∗p < 0.01, ∗∗∗p < 0.001, ∗∗∗∗p < 0.0001, A-I) ∗ chow vs. DTS,  chow vs. NTS,  DTS vs. NTS; 2-way ANOVA: A) time p < 0.0001, group p < 0.01, interaction p > 0.05; B) time, interaction p < 0.05, group p > 0.05; C) time, group p < 0.001, interaction p < 0.05; D) time p < 0.001, group, interaction p > 0.05; E) time p < 0.0001, group p > 0.05, interaction p < 0.001; F) time p < 0.0001, group, interaction p > 0.05; G) time, interaction p < 0.0001, group p < 0.001; H) time, group, interaction p < 0.0001; I) time, interaction p < 0.0001, group p < 0.01. Mixed-effects analysis: J) time, time x group p < 0.0001, group p < 0.01; K) time p < 0.0001, group p < 0.05, time x group p > 0.05; L) time, time x group p < 0.0001, group p < 0.01. Data are shown as mean ± SEM; A) n = 5–6, B–C) n = 4–6, D-I) n = 4–6, J-L) n = 6–8 per ZT and group. DTS: daytime snack, NTS: nighttime snack, R: rhythmic, NR: not rhythmic.

As we observed snack-induced changes in rhythmic energy expenditure and increased plasma lipid levels at ZT3 (Figure 2, Figure 3, Suppl. Figures A.3-4), we next investigated whether daily energy equivalents in glucose and TAG were altered. To address this question, we investigated serum parameters across the day after six weeks of snacking (Figure 6A–C). Serum glucose levels were comparable in chow and NTS mice; however, NTS mice had a slight elevation around ZT21 which in contrast to the chow group resulted in non-rhythmic glucose levels (Figure 6A). To the contrary, serum glucose levels were rhythmic in DTS mice and significantly increased at ZT9 and 15 compared to the NTS group (Figure 6A). Notably, serum glucose levels were not increased at ZT3 – 1 h after the DTS – which is why we investigated the serum insulin concentration. Despite showing high variation within the time points per group, insulin levels were increased in the DTS compared to the NTS group (Figure 6B). Insulin levels in the NTS group were rhythmic (Figure 6B). TAG levels were comparable between the chow and NTS groups but elevated in the DTS group at ZT3 (Figure 6C). No rhythmicity was detected for TAG levels (Figure 6C). Due to the changed energy equivalents, we then hypothesized that snacking would affect the nutrient uptake and the circadian clock in the small intestine. To address this question, we analyzed gene expression in the jejunum of mice after six weeks on snacking. Glucose transporter 2 (GLUT2) and sodium/glucose cotransporter 1 (SGLT1), encoded by *Slc2a2* and *Slc5a1*, are responsible for intestinal glucose absorption whereby SGLT1 is located on the apical and GLUT2 on the basolateral side of the enterocyte [32]. Other studies also report apical GLUT2 expression [33]. We observed comparable rhythmic gene expression of *Slc2a2* and *Slc5a1* between the chow and NTS groups in the jejunum (Figure 6D–E). *Slc2a2* expression was increased at ZT3 and *Slc5a1* expression reduced at ZT9 in the DTS group compared to the NTS and chow groups (Figure 6D–E). Diacylglycerol acyltransferases 1 and 2 (DGAT1/2) are synthesizing new TAG, whereby DGAT2 is especially involved in hyperglycaemic conditions [34]. *Dgat2* expression was not significantly different between the three groups, but *Dgat2* expression became arrhythmic in the DTS group (Figure 6F).

In the duodenum, we observed comparable rhythmic gene expression of *Slc2a2* and *Slc5a1* between the chow and NTS groups in duodenum (Suppl. Figure A.6A-B). In the DTS group *Slc2a2* and *Slc5a1* gene expression was rhythmic but reduced at ZT9 compared to the NTS or chow group, respectively (Suppl. Figure A.6A-B). *Dgat2* expression was increased at ZT3 in the DTS group compared to the chow and NTS groups, but it was reduced at ZT15 under both snacking conditions (Suppl. Figure A.6C). Additionally, *Dgat2* expression lost rhythmicity in the DTS group (Suppl. Figure A.6C). We did not observe major snack-induced changes in expression of *Dgat1* or *CD36* in duodenum or jejunum and gene expression was mostly arrhythmic (Suppl. Figure A.7).

While gene expression of *Bmal1*, *Dbp*, and *Nr1d1* was similar between the chow and NTS groups in the jejunum and duodenum, clock gene expression was dampened in the DTS group in both tissues (Figure 6G–I, Suppl. Figure A.6D-F) indicating that chronic mistimed snacking is disturbing intestinal clock gene expression.

Due to the changes in expression of genes involved in nutrient uptake along with reduced energy expenditure at night and two daily peaks in locomotor activity and core body temperature in the DTS group (Figure 2, Figure 4, Figure 6, Suppl. Figure A.3), we hypothesized that DTS mice would reabsorb more energy. Similar to food intake after 1.5 weeks on snack (Figure 1K), energy intake was comparable between chow and NTS mice in the first 6 h of the rest and inactive phase after 5 weeks on snacking (Figure 6J). In contrast, daytime snacking increased energy intake from ZT0-6 and decreased energy intake from ZT12-18 compared to the chow and NTS groups (Figure 6J). While excreted energy was comparable from ZT0-6 in all three groups, it was reduced in the NTS compared to the chow group from ZT12-18 (Figure 6K). The assimilated energy from ZT0-6 was comparable between the chow and NTS groups but increased in DTS mice compared to both other groups (Figure 6L). From ZT12-18, assimilated energy was decreased in DTS mice compared to the chow and NTS groups but increased in NTS mice compared to the chow group (Figure 6L).

Together, these data indicate differences in energy equivalents during the 24-hour day and a disrupted uptake of nutrients in the small intestine in line with a dampening of intestinal clock gene expression after daytime snacking. We could further show increased total assimilated energy in the DTS group which – in line with our data on energy expenditure, activity and temperature – could explain the observed body weight phenotype.

### 3.6 The effect of snacking on body weight development requires a functional circadian clock under constant darkness conditions

**Figure 7:**
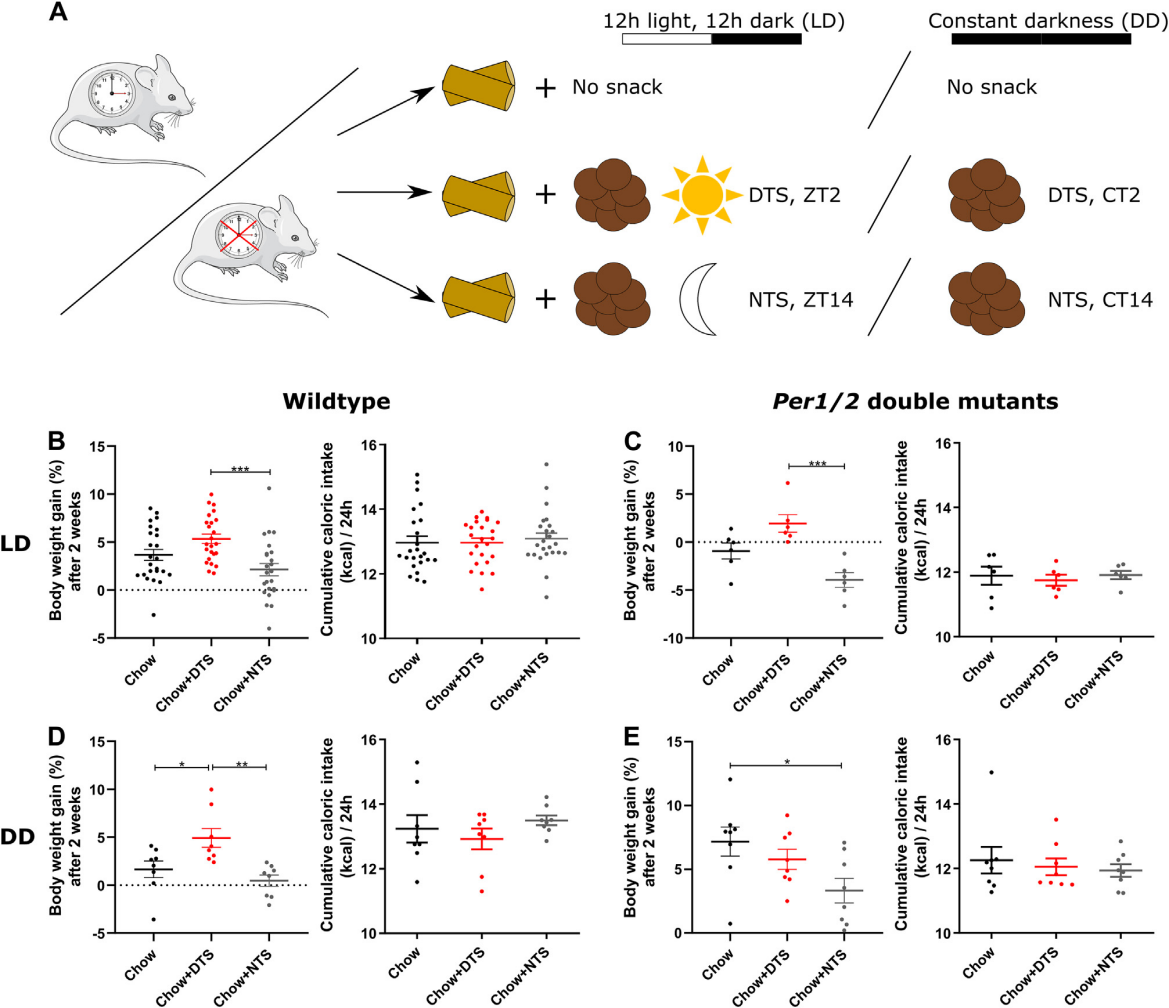
**Snacking requires a functional circadian clock in constant darkness conditions.** A) Experimental setup for wildtype and clock deficient *Per1/2* double mutant mice housed under standard 12h:12h light–dark conditions (LD) as well as in constant darkness (DD). Mice were separated in three groups receiving either (i) no snack (control group), or (ii) a daytime snack (DTS) at *zeitgeber* time (ZT) 2 (in LD) or at circadian time (CT) 2 (subjective DTS in DD), or (iii) a nighttime snack (NTS) at ZT14 (in LD) or at CT14 (subjective NTS in DD). B-E) Body weight gain in % after two weeks on snacking regimen as shown in (A) normalized to the body weight on the day before snacking was started and cumulative caloric intake per 24 h. Body weight gain and cumulative caloric intake per 24 h of B) wildtype (n = 24), C) *Per1/2* double mutants (n = 6) in LD and of D) wildtype (n = 8), E) *Per1/2* (n = 8) double mutants in DD. B-E) Bonferroni post-hoc test ∗ p < 0.05, ∗∗p < 0.01, ∗∗∗p < 0.001; 1-way ANOVA body weight gain: B) p < 0.001; C) p < 0.001; D) p < 0.01; E) p < 0.05; 1-way ANOVA cumulative caloric intake per 24 h: B-E) p > 0.05. Data are shown as mean ± SEM. Mouse image: smart.servier.com.

We next sought to investigate whether the effect of chronic daytime snacking on body weight development require a functional circadian clock. We therefore performed the snacking experiment over two weeks in a 12h:12h light–dark cycle (LD) as well as in constant darkness (DD) in wildtype and clock-deficient *Per1/2* double mutant mice (Figure 7A). Data for the wildtype mice in LD after two weeks on snacking were taken from the same experiment as in Figure 1B. Compared to the wildtype control group, DTS mice slightly gained and NTS mice slightly reduced weight in LD resulting in a significant increase in body weight gain in the DTS compared to the NTS group (Figure 7B). In *Per1/2* double mutant mice in LD body weight gain was slightly increased in the DTS mice compared to the control group, however, this change was not significant (Figure 7C). In contrast, NTS *Per1/2* double mutant mice reduced body weight in LD resulting – in line with the wildtype mice in LD – in a significant difference between the NTS and DTS groups (Figure 7C). In DD, wildtype DTS mice still gained more body weight compared to the control and NTS groups while NTS mice were comparable to the control group (Figure 7D). *Per1/2* double mutant DTS mice did not show increased body weight gain anymore in DD, they rather reduced body weight; however, this change was not significant (Figure 7E). Interestingly, *Per1/2* NTS mice significantly reduced body weight compared to the control group in DD despite of snacking (Figure 7E). Daily cumulative caloric intake was comparable between the groups in the four experimental cohorts, *i.e.*, wildtype and *Per1/2* double mutants in LD and DD (Figure 7B–E). *Per1/2* double mutants consumed on average 1 kcal less than wildtype mice under both housing conditions (Figure 7B–E). FAA and increased activity around the snacking time were observed in wildtype DTS mice in LD and DD, whereas nighttime snacking strengthened the normal activity rhythm (Suppl. Figure A.8). In *Per1/2* double mutants, diurnal activity rhythms were observed under LD conditions with FAA in the DTS group and increased activity around the snacking time in the NTS group (Suppl. Figure A.8). Activity rhythms were abolished in *Per1/2* double mutants housed under DD conditions, but mice were still more active around their respective snacking time (Suppl. Figure A.8). In summary, these data suggest that the effect of chronic daytime snacking increasing body weight gain requires the functionality of the circadian clock at least under DD conditions.

## 4 Discussion

In this study we focused on the effect of snack timing on body weight development and metabolic homeostasis. Our results show that chronic snacking in the beginning of the rest phase increases body weight gain and fat mass by disrupting food intake as well as metabolic rhythms in male mice. In contrast, mice fed a timed snack in the early active phase were protected from the body weight effect and rhythm disruptions. Notably, our data suggest that the effect of chronic daytime snacking on body weight development is requiring a functional circadian clock at least under DD conditions.

In our chronic snack experiment we observed an effect of snack timing on body weight gain and food intake rhythms (Figure 1). Mice consuming a daily snack in the rest phase over six weeks gained body weight and fat mass. These body weight data are in line with data in rats showing that chocolate in the early rest phase increases body weight whereas chocolate snacking in the early active phase was even reducing body weight [17]. In humans, the risk of developing obesity is increased with high-calorie consumption in the evening [35]. We found that DTS mice did not gain weight due to changes in cumulative caloric intake, but their food intake rhythms were disrupted. Daily food composition was comparable between DTS and NTS mice but was distinct at different times of the day in the snacking groups (Figure 1, Suppl. Figure A.2). Palatable diets such as a high-fat diet are known to increase food intake during the rest phase and that it is rather the time-of-day when the calorie-dense food is consumed than the caloric value *per se* [1,2].

In line with chow intake rhythms under *ad libitum* conditions, energy expenditure and RER peak in the beginning of the active phase [4,12]. We did not expect a large effect on energy expenditure and RER in the NTS group as the NTS strengthens the normal feeding rhythm. In contrast, DTS mice showed reduced energy expenditure and RER in the active phase as well as a second RER peak, indicating carbohydrate metabolism, in the rest phase (Figure 2, Suppl. Figure A.3). These results are comparable to data of light fed mice that expend less energy throughout the dark phase but the RER of those mice is completely inverted in line with their food intake [12]. Notably, we observed an increased RER in the early inactive phase in DTS mice, although their diet consisted of less carbohydrates compared to the chow group (Figure 2, Figure 1K). This might be explained by the altered chow intake rhythm in DTS mice (Suppl. Figure A.2B) as they consume most of the chow prior to the snack which increases the normal amount of available carbohydrates at this time of day and thus might impact RER. Although total 24 h energy expenditure was not significantly reduced in DTS mice compared to the NTS and chow groups, the clear reduction of energy expenditure throughout the active phase adds on the explanation of the body weight phenotype in the DTS group.

Due to the observed changes in energy expenditure and RER, we hypothesized that lipids would be increased in the daytime snack group. We confirmed this by our lipidomics study showing overall increased plasma lipid levels at ZT3 after daytime snacking (Figure 3, Suppl. Figure A.4). We identified the main lipid classes in cluster 1 and 2 and summarized them as they both show snack-induced changes (Figure 3). Elevated TAG as well as DAG levels could originate from dietary fats, e.g. TAGs and DAGs are contained in cocoa butter [36,37]. Apart from dietary fats, increased DAG levels could also result from TAG or phospholipid hydrolysis as well as from sphingomyelin synthesis [38]. Increased TAG levels disturb insulin-dependent glucose transporter 4 (GLUT4) signaling and could thereby add on changes in glucose metabolism which was recently suggested to contribute to lipidomic and metabolomic changes in a rat model of shift work with light-phase restricted feeding [39]. Phosphoinositides, the precursor of PIs, were reported to be involved in insulin signaling [40,41]. PIs have an anti-obesogenic effect when accumulating in liver of HFD fed mice [42]. Phospholipids are important for lipoprotein formation and stability [43]. In particular, PCs are involved in TAG transport by chylomicron as well as VLDL assembly and secretion and PEs in lipid droplet fusion coordinated by the amount of PEs on the lipid droplet surface [43]. Specifically, the product of PC hydrolysis, LPC, was suggested to activate the pathway of prechylomicron output and to promote lipid uptake [44,45]. In our study LPC was not significantly increased but we observed a slight elevation in the DTS group at both time points (p = 0.053). Our data show increased sphingomyelin and ceramide levels in the DTS group at ZT3. Sphingomyelins are synthesized out of ceramide using PCs creating DAG as a byproduct but this reaction is bidirectional so that sphingomyelins can be used to produce ceramides [46,47]. In context of high-fat diet sphingolipid metabolism, sphingomyelins were suggested to serve as a pool for ceramide synthesis [48]. Additionally, inflammation in obesity is inducing enzymes required for *de novo* ceramide synthesis [49]. Ceramides have an antagonistic effect on glucose uptake via GLUT4 and is known to inhibit Akt signaling [[49], [50], [51]]. The increased lipids in the DTS group seem to increase lipid uptake from the intestine and interfere with glucose homeostasis. Interestingly, it was recently published that an oral glucose load would mobilize TAG from enterocyte storages enhancing chylomicron secretion in humans [52]. This is in line with our data showing increased serum glucose levels (Figure 6) and further support increased lipid transport from the intestine through chylomicron transport. We observed increased CE in our snacking mice suggesting dyslipidemia which was further supported by total serum cholesterol levels and cholesterol from HDL and LDL/VLDL lipoprotein fractions that were elevated in the DTS group (Figure 3, Suppl. Figure A.4).

Compared to chow mice we found reduced locomotor activity throughout the second half of the active phase as well as FAA and increased activity around the snacking time in the DTS group (Figure 4). In the NTS group activity was condensed and increased during the first half of the active phase, *i.e.,* around their snacking time. In general, mice develop FAA to a timed expected meal [53]. Specifically, a timed daytime chocolate snack is also known to induce FAA in mice [54]. In women, a chronic evening chocolate consumption caused increased activity afterwards [18]. In line with induced locomotor activity, the daily core body temperature rhythm was strengthened in NTS mice whereas, in the DTS group, body temperature was reduced in the second half of the active phase and elevated in the first half of the rest phase (Figure 4). A moderate increase in pre-prandial thermogenesis as well as FAA has previously been shown in mice fed a timed high-fat meal [55]. Postprandial thermogenesis after a daily timed chocolate snack in the early active phase was reported in rats, however, a chocolate snack in the early inactive phase did not induce thermogenesis in this study [17]. Locomotor activity around the snacking time in the DTS and NTS group increased over the course of the experiment whereas body temperature in the DTS group directly reached a plateau on the first day of snacking but was comparable during the first 6 h of the active phase (Figure 5). Our data are consistent with previous studies showing that FAA develops within one week [56]. We expected a higher temperature increase at ZT0-6 compared to ZT12-18 as the capacity for a temperature increase in mice is higher during daytime. These data are in line with a temperature upregulation after acute single snacking at daytime in mice [57].

Because of our finding of increased body temperature in the DTS group we investigated iBAT temperature. iBAT surface temperature was increased in DTS compared to NTS as well as the inner-ear temperature as a readout of core body temperature (Suppl. Figure A.5). As these data are resulting in an unchanged maximal iBAT surface to inner-ear temperature ratio in comparison to the chow and NTS groups and tail temperature was not elevated at the same time, DTS mice deliberately have a higher body temperature compared to NTS mice which they are not counteracting by heat dissipation via the tail. This indicates that DTS mice are not hyperthermic but rather defend a higher body temperature set-point (pyrexia) around the snacking time. Although increased locomotor activity contributes to the increase in core body temperature, as mice start being active before the snacking time, a role of BAT thermogenesis in body temperature regulation after the postprandial phase and activity period cannot be ruled out at this point as body temperature stays elevated for 6 h after the snack.

In our study we found increased serum glucose, insulin, and TAG levels in the DTS group (Figure 6). Increased serum glucose levels indicate hyperglycemia that could promote type 2 diabetes and obesity. Interestingly, serum glucose levels were not elevated 1 h after the DTS. In contrast, serum insulin was increased in the DTS compared to the NTS group and might therefore counteract the increased glucose levels 1 h after the DTS. Potentially, glucose is already increased around the snacking time due to elevated chow consumption stimulating insulin secretion that might then react faster to reduce serum glucose after the snack. Increased serum TAG levels at ZT3 in the DTS group were – in line with our lipidomics screen (Figure 3) – pointing at hypertriglyceridemia.

We found dampened clock gene expression in the intestine after daytime snacking as well as altered expression of genes involved in nutrient utilization suggesting altered uptake of glucose and TAG (Figure 6). Food is the dominant *zeitgeber* for clocks in peripheral tissues leading to an uncoupling of peripheral tissue clocks from the SCN clock after light-phase restricted feeding [9]. We did not expect a large phase shift in clock gene expression in the DTS group as we provided a timed snack, and the mice still had food access in the active phase. We did expect a dampening in intestinal clock gene expression as also long-term high-fat diet feeding leads to dampening in clock gene expression in, e.g., fat and liver [1]. Intestinal clock gene expression is linked to the development of obesity. Mice deficient of *Nr1d1* absorb more dietary fat which promotes high-fat diet induced obesity [58]. This is in line with our chronic snack experiment where *Nr1d1* expression is reduced and plasma lipids increased at ZT3 in the DTS group. BMAL1 is known to regulate *Dgat2* transcription [58] which could explain that – in line with *Bmal1* – *Dgat2* expression is in general downregulated in the intestine. Duodenal *Dgat2* expression is upregulated in the DTS group at ZT3 compared to the control and NTS groups which might TAG synthesis at that time point (Suppl. Figure A.6). Hypertriglyceridemia can be induced by carbohydrates and DGAT2 was discussed to link glycaemia and triglyceridemia [34]. Notably, the duodenum and proximal jejunum are involved in lipid absorption in enterocytes [59]. As we used the middle part of the jejunum for our analysis, we expected effects in lipid absorption rather in the duodenum than in the jejunum. Diet is known to influence the microbiome. High-fat diet feeding shifts the microbiome towards bacterial species that promote lipid absorption and it was shown that these species increase among others *Dgat2* expression [60]. We were not feeding a high-fat diet, but the snacking groups had slightly more fat in their diet compared to the control group. We speculate that a DTS might change the microbiome and thereby promoting the body weight phenotype. The jejunum is most important for glucose uptake whereby mainly SGLT1 (encoded by *Slc5a1*) transports glucose from the intestinal lumen to the enterocyte and GLUT2 (encoded by *Slc2a2*) from the enterocyte into the blood stream [61]. However, GLUT2 might adjust glucose transport by translocation to the apical membrane increasing glucose uptake into the enterocyte [[61], [62], [63]]. We found a downregulation in *Slc5a1* expression at ZT9 in duodenum and jejunum, whereas *Slc2a2* expression was upregulated at ZT3 in the jejunum in the DTS group. These data suggest a dysregulation of glucose uptake that might be caused by disturbed clock gene expression in the duodenum and jejunum. It was recently shown that the intestinal clock is important for glucose absorption [64]. In our daytime snacking mice, an upregulation of *Slc2a2* expression might compensate for changes in *Slc5a1* expression and explain the increased serum glucose levels.

Additionally, we observed changes in intestinal resorption after DTS. DTS mice had an increased assimilated energy from ZT0-6 but slightly decreased assimilated energy from ZT12-18 compared to the chow and NTS groups (Figure 6). The overall increase in assimilated energy in the DTS group as well as more assimilated energy in the inactive phase could add an explanation to the body weight phenotype in our DTS mice. In contrast, assimilated energy was increased in the NTS group compared to the chow and DTS groups during the first half of the active phase supporting the natural rhythm. Notably, we observed negative values of assimilated energy indicating that mice excreted more energy than they took in during the investigated time interval and were using stored energy. Our study is limited as we did not investigate time intervals from ZT6-12 and 18–24. We speculate that in line with food intake (Figure 1K) assimilated energy would be rather comparable between the three groups from ZT6-12 but might be reduced in the DTS compared to the chow and NTS groups from ZT18-24. This might influence daily 24h-assimilated energy, but the DTS mice still reabsorbed more energy at unfavorable times of the day, *i.e.* in the beginning of the normal inactive phase.

Our results indicate that the effect of chronic daytime snacking on body weight regulation requires the functionality of the circadian clock at least under DD conditions (Figure 7). Wildtype DTS mice still showed body weight gain in DD with a pattern comparable to wildtype mice in LD. Overall, the effect of body weight gain was slightly reduced in wildtype mice in DD. DTS *Per1/2* double mutant mice still had a higher body weight gain in LD, however, *Per1/2* double mutants in general rather reduce weight during the two experimental weeks. 24-hour cumulative caloric intake was comparable between the groups in all experimental cohorts. *Per1/2* double mutant mice show nocturnal activity patterns in LD but become arrhythmic in DD [20]. In *Per1/2* double mutant mice in DD the snacking effect on body weight development was abolished suggesting that at least under DD conditions a functional circadian clock is required. The absence of a functional circadian clock and the external signal light in *Per1/2* double mutants abolishes food intake and locomotor activity rhythms [20]. Consequently, there was no feeding rhythm that could be disturbed in our experimental setup. NTS mice even reduced body weight compared to the controls suggesting that a snack fed at the correct time of the day (NTS) is beneficial for body weight regulation. We speculate that the contribution of the circadian clock to the body weight effect might be stronger than the potential masking effect of light and food as body weight gain was rather mild in *Per1/2* double mutants in LD.

In conclusion, we show that chronic daytime snacking increases body weight gain and has disrupting effects on metabolic rhythms. Additionally, our data suggest that chronic daytime snacking dampens intestinal clock gene expression and changes nutrient uptake and utilization that together with increased assimilated energy promotes a body weight phenotype. An experimental setup in clock mutant mice as well as in constant darkness indicates that the observed effects require a functional circadian clock or a rhythmic masking stimulus leading to rhythmic food intake. Our study is limited to male mice but it would be interesting to investigate the effect of chronic daytime snacking in females as sex differences in dopamine D1 receptor mediated food anticipation are known [65]. Additionally, it was shown that estrogen influences the adipocyte clock protecting female mice from high lipid accumulation under rhythm disruptions such as jetlag [19]. As our data indicate an effect of the circadian clock on body weight regulation, future studies are needed to investigate the molecular underpinnings of this regulation. As the snacking effect on body weight regulation persisted in clock-deficient *Per1/2* double mutant mice, we speculate that a potential masking effect of light and food on rhythmic behavior might contribute body weight regulation. Our observation in mice could be useful for behavioral therapies of body weight regulation and nutritional regimens.

## Data Availability

Data will be made available on request.
