# Supplementary material for: Rest phase snacking increases energy resorption and weight gain in male mice
Source: Mol Metab. 2023 Feb 4;69:101691. doi: 10.1016/j.molmet.2023.101691 (PMC9950950; doi:10.1016/j.molmet.2023.101691)
Supplement: Multimedia component 1 [file mmc1.docx]

**
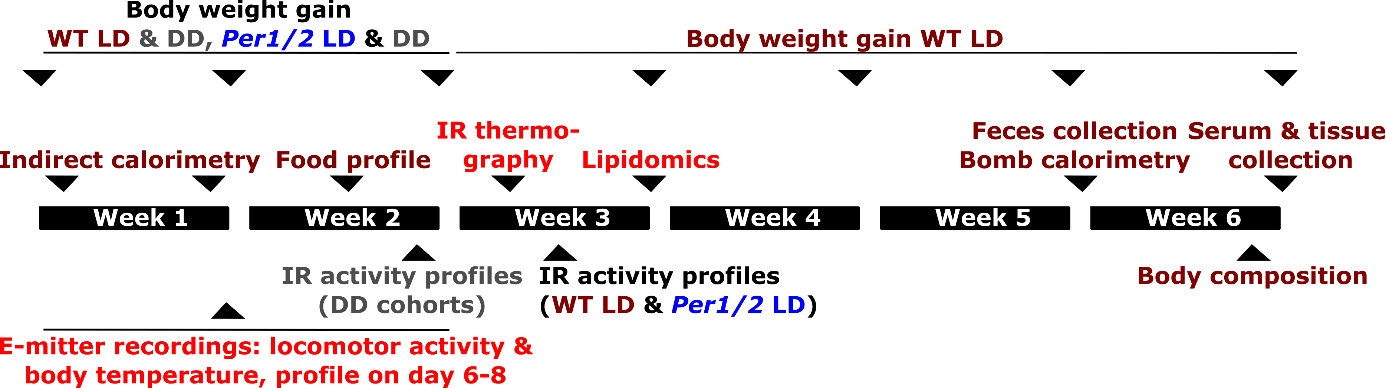
**

**A.1. Timeline figure.** In this timeline all measurements during the weeks of snacking are indicated. Body weight gain was measured in all cohorts before the snacking was started and monitored for two weeks or over six weeks in the wildtype (WT) cohort housed under 12:12 light-dark conditions (LD), respectively. In the WT/LD cohort, indirect calorimetry was performed in the first week, a food profile after 1.5 weeks on snacking, and an IR locomotor activity profile after 2.5 weeks on snacking. Feces for bomb calorimetry were collected after five weeks. Body composition was measured towards the end of week six on snacking and serum and tissues were collected at the end of the experiment. In another WT/LD cohort locomotor activity and body temperature were recorded for the first two weeks on snacking using E-mitters. Profiles were analysed from day 6-8 on snacking. IR thermography was performed with these mice in the third week of snacking and at the end of this week plasma was collected for an untargeted lipidomics screen. Infrared (IR) activity profiles of the *Per1/2* double mutant/LD cohort were recorded after 2.5 weeks on daily snacking. IR activity profiles of the WT and *Per1/2* double mutant cohort housed under constant darkness (DD) conditions were recorded at the end of the second snacking week.

**
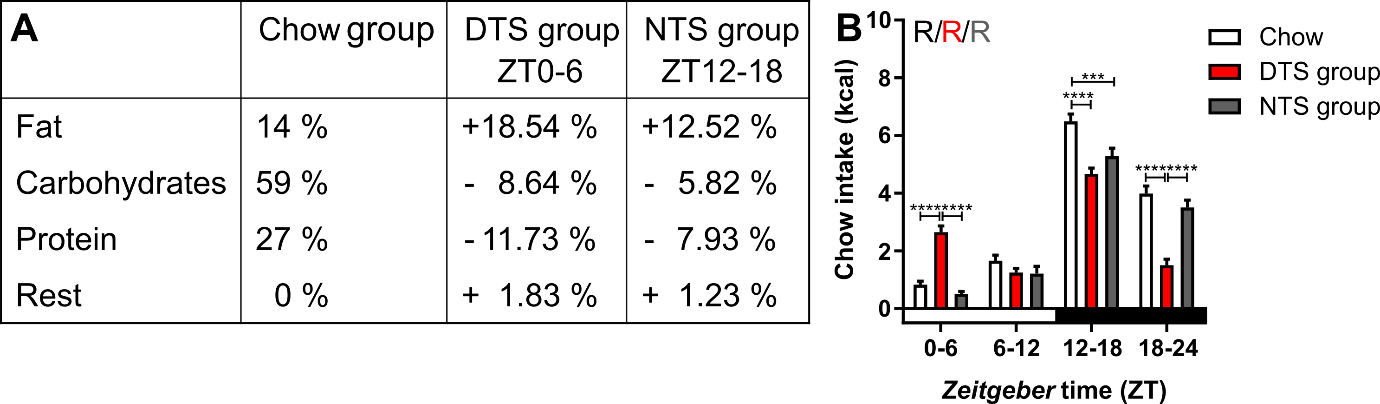
**

**A.2. Chow intake rhythms and food composition during snacking times.** A) Food composition at intervals during snacking time (daytime snack (DTS) group ZT0-6, nighttime snack (NTS) group ZT12-18) in comparison to the chow group. B) Chow intake after 1.5 weeks on snack ad food profile in 6h-resolution, R: rhythmic, Bonferroni post-hoc test *** p<0.001, **** p<0.0001; Mixed-effects analysis: time, group, time x group p<0.0001. Data are shown as mean ± SEM; n=22-24 per ZT and group.


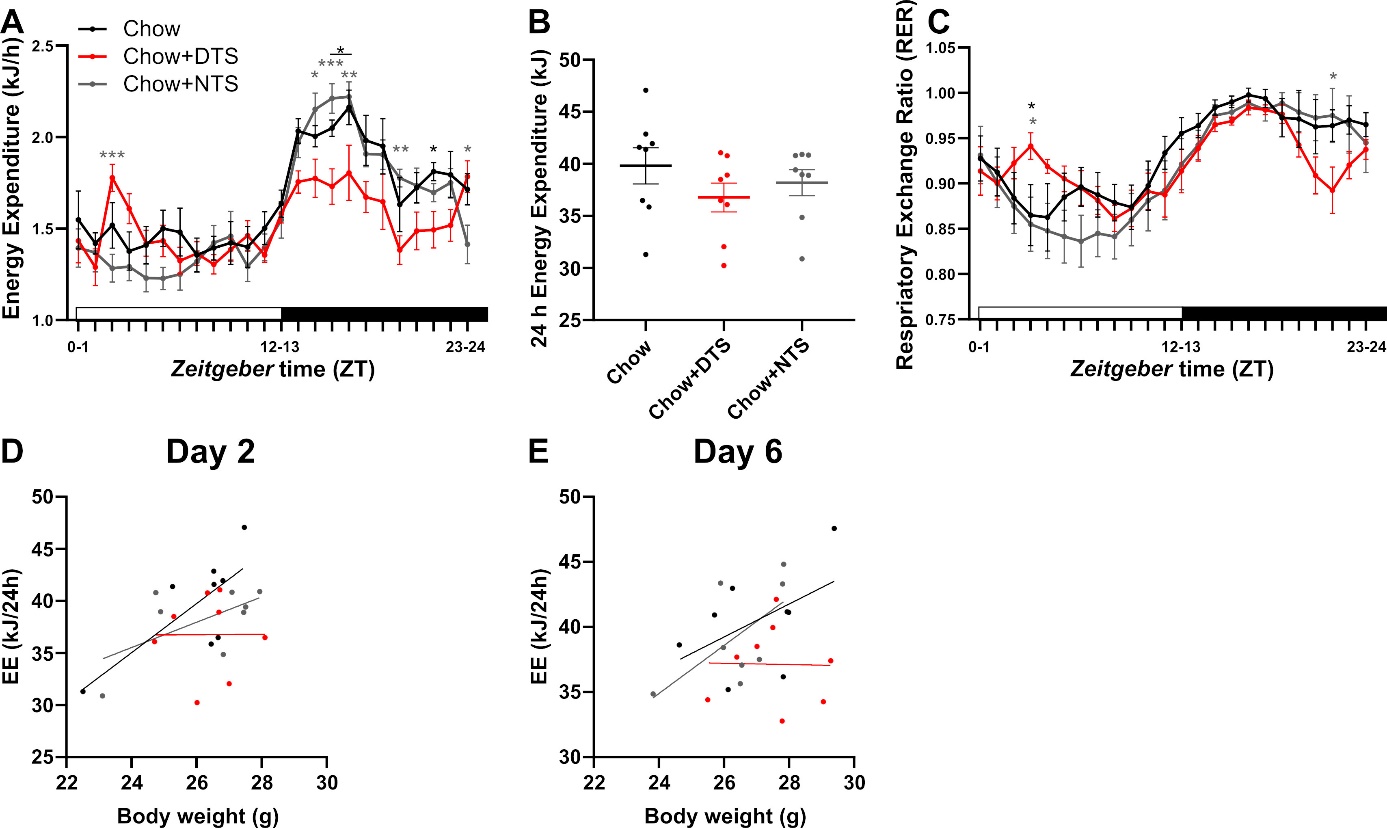


**A.3. Daytime snacking alters daily metabolism at the second day of snacking.** A) Energy expenditure. B) Total daily (24 h) energy expenditure. 1-way ANOVA: p>0.05. C) Respiratory exchange ratio (RER). D-E) Energy Expenditure (EE) vs. body weight on D) day 2 and E) day 6 of snacking. ANCOVA analysis using body weight as co-variant revealed no significant differences in energy expenditure between the groups (p=0.084); A, C) Bonferroni post-hoc test * p<0.05, ** p<0.01, *** p<0.001. **** p<0.0001, * chow vs. DTS, * chow vs. NTS, * DTS vs. NTS; 2-way ANOVA: time p<0.0001, group p>0.05, interaction A) p<0.0001, C) p<0.01. A-C) Data are shown as mean ± SEM; A-E) n=8. DTS: daytime snack, NTS: nighttime snack

**
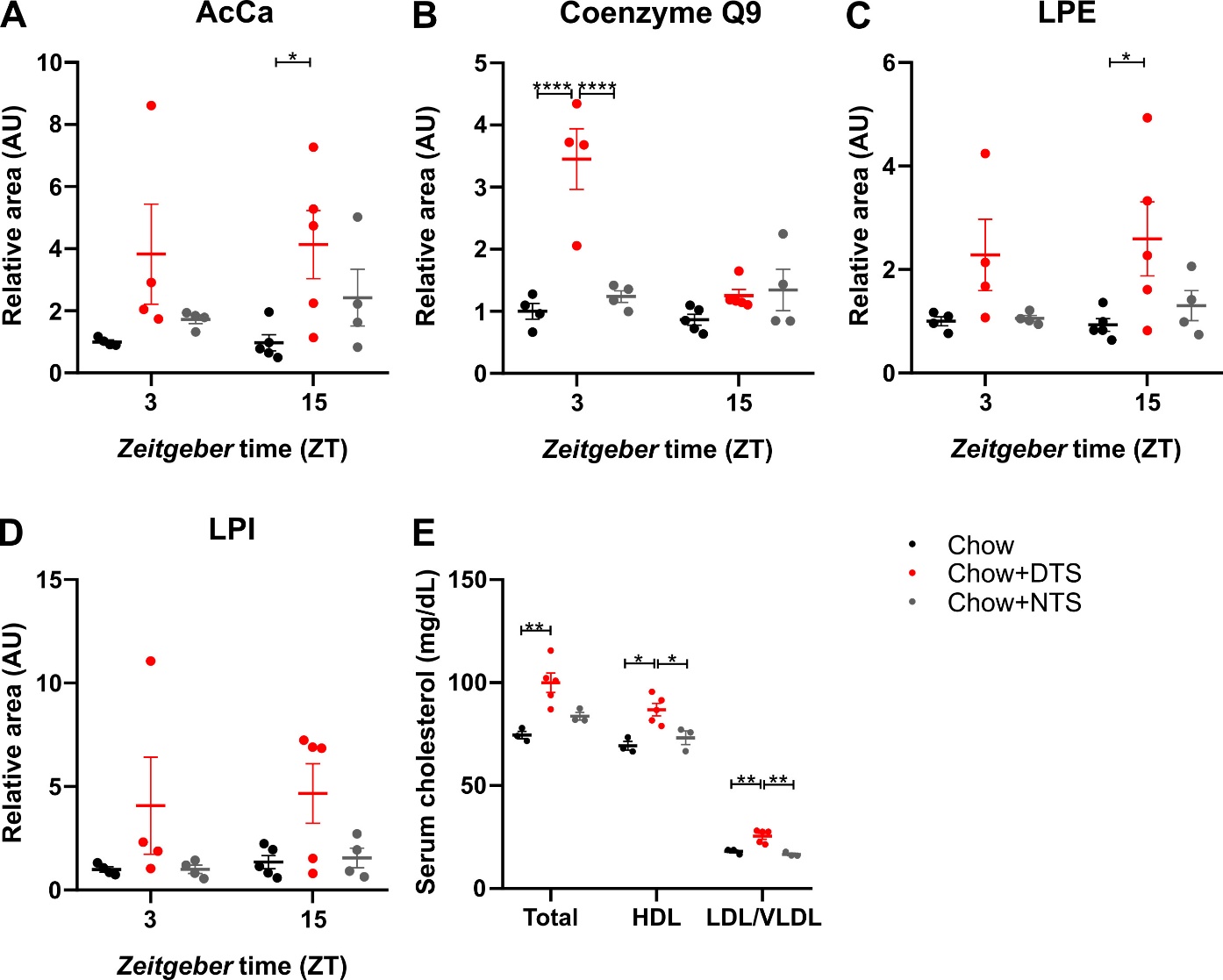
**

**A.4. Lipidomics analysis.** A-D) Lipids detected in plasma after three weeks on snacking at *zeitgeber* time (ZT) 3 and 15 during the lipidomics screen grouped in their respective lipid classes. Single lipids that were detected during the screen are listed in supplemental Table B. A) Acylcarnitines (AcCA), B) Coenzyme Q9, C) Lysophosphatidylethanolamines (LPE), and D) Lysophosphatidylinositols (LPI). Further lipid classes are presented in Figure 6. E) Total serum cholesterol and cholesterol from lipoprotein fractions. A-E) Bonferroni post-hoc test * p<0.05, ** p<0.01, **** p<0.0001; A-D) 2-way ANOVA: A) time p>0.05, group p<0.01, interaction p>0.05; B) time p<0.01, group p<0.0001, interaction p<0.001; C) time p>0.05, group p<0.01, interaction p>0.05; D) time p>0.05, group p<0.05, interaction p>0.05. E) 1-way ANOVA: total, HDL, LDL/VLDL p<0.01. Data are shown as mean ± SEM; A-D) n=4-5, E) n=3-5. AU: arbitrary units, DTS: daytime snack, HDL: high density lipoprotein, LDL: low density lipoprotein, NTS: nighttime snack, VLDL: very low density lipoprotein


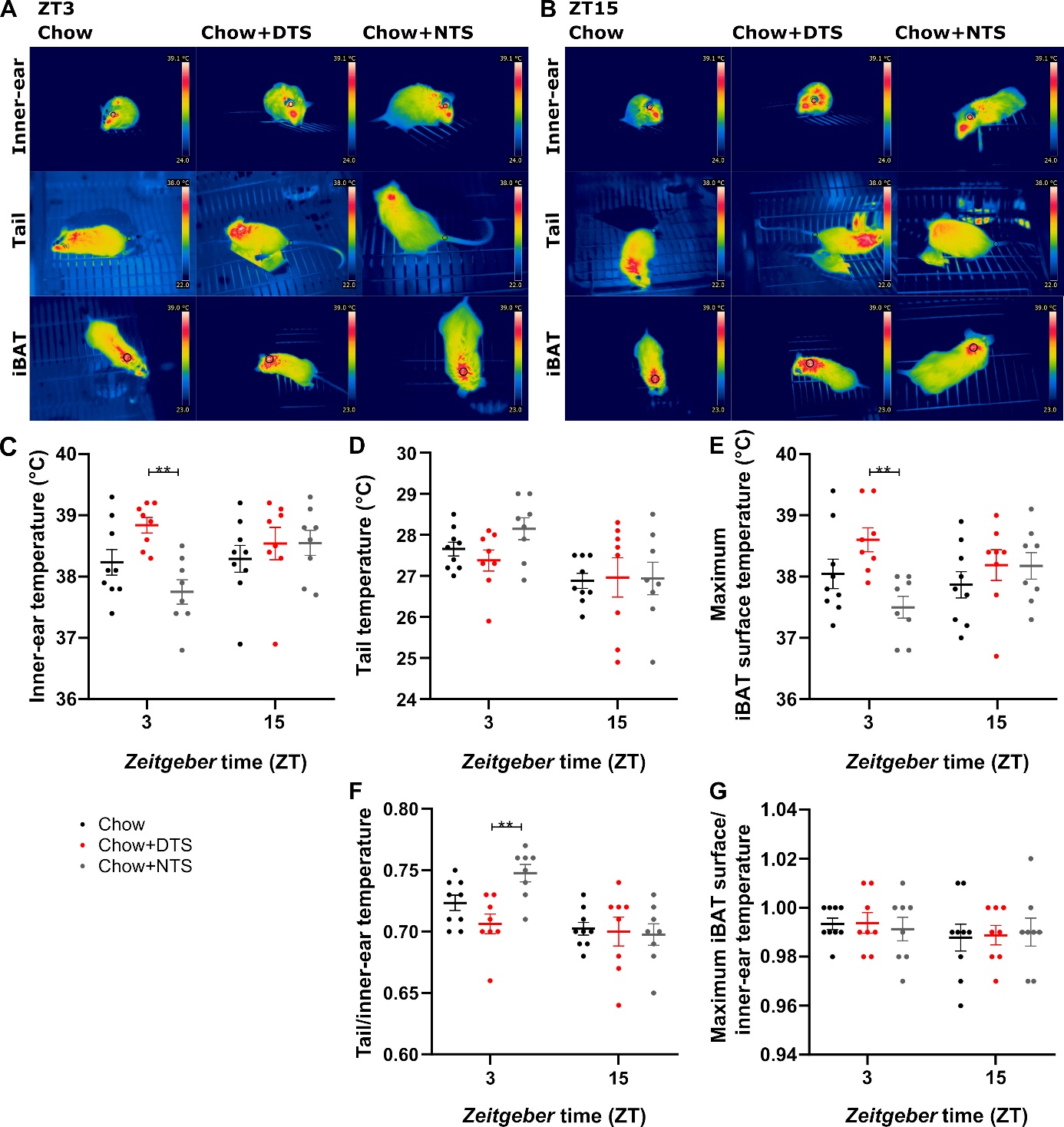


**A.5. Daytime snacking alters the regulation of body temperature during the rest phase.** Infrared thermography pictures of inner-ear, tail, and interscapular brown adipose tissue (iBAT) temperature of chow, daytime snack (DTS) and nighttime snack (NTS) mice at A) *zeitgeber* time (ZT) 3 and B) ZT15 in the third snacking week. C) Inner-ear temperature. D) Tail temperature. E) Maximum iBAT surface temperature. F) Ratio of tail and inner-ear temperature. G) Ratio of maximum iBAT surface and inner-ear temperature. C-G) Bonferroni post-hoc test ** p<0.01; 2-way ANOVA: C) time, group p>0.05, interaction p<0.01; D) time p<0.001, group, interaction p>0.05; E) time, group p>0.05, interaction p<0.01; F) time p<0.001, group p>0.05, interaction p<0.05; G) time, group, interaction p>0.05. Data are shown as mean ± SEM; n=8-9.


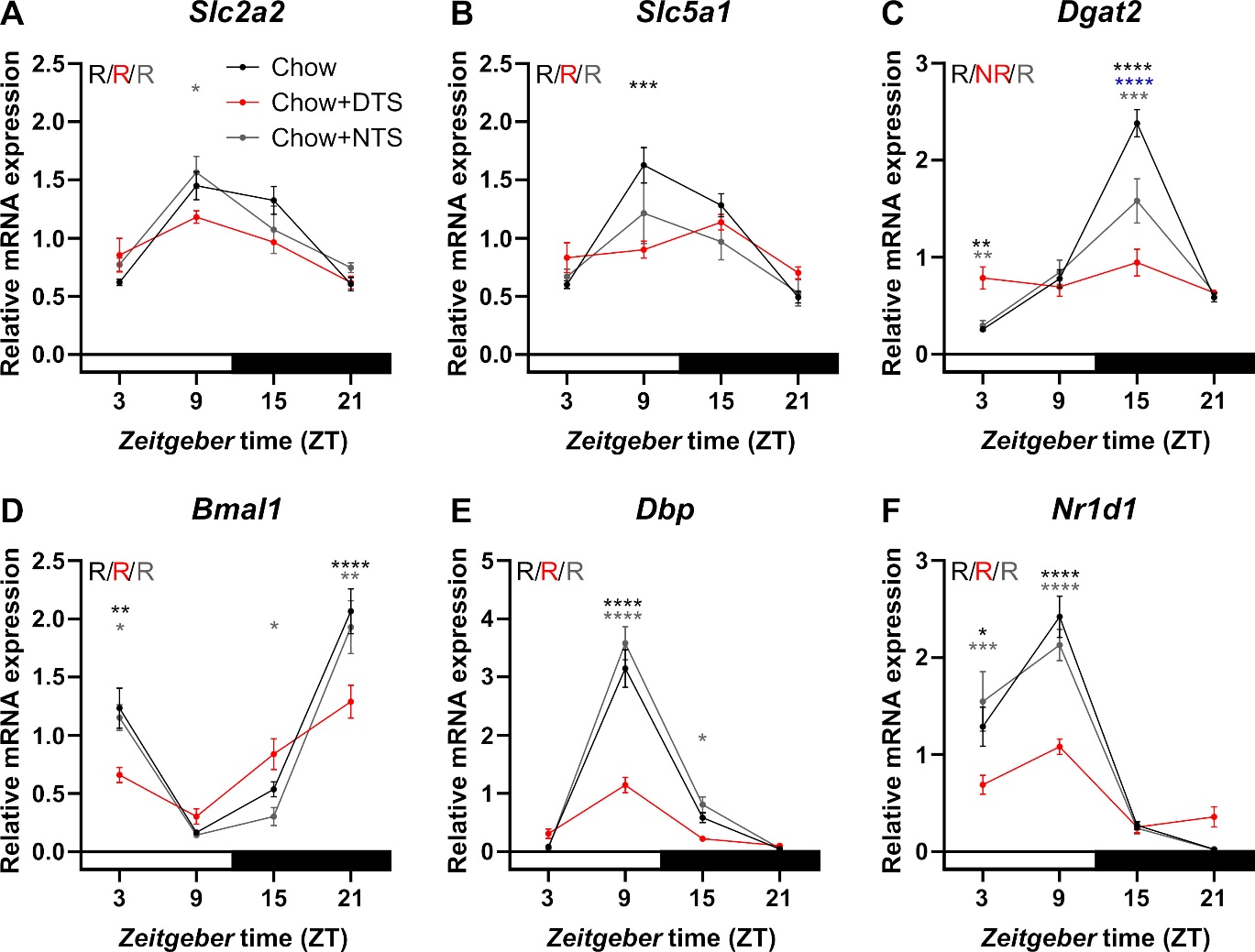


**A.6. Daytime snacking alters expression of clock genes and genes involved in nutrient utilization in the duodenum.** Expression of glucose uptake transporter genes A) *Slc2a2*, B) *Slc5a1*, C) *Dgat2* and clock genes D) *Bmal1*, E) *Dbp*, F) *Nr1d1* in the duodenum after six weeks on snacking. Bonferroni post-hoc test * p<0.05, ** p<0.01, *** p<0.001, **** p<0.0001, * chow vs. DTS, * chow vs. NTS, * DTS vs. NTS; 2-way ANOVA: A) time p<0.0001, group p>0.05, interaction p<0.05; B) time p<0.0001, group p>0.05, interaction<0.01; C) time, interaction p<0.0001, group p<0.01; D) time, interaction p<0.0001, group p<0.05; E) time, group, interaction p<0.0001; F) time, interaction p<0.0001, group p<0.001. Data are shown as mean ± SEM; n=4-6 per ZT and group. DTS: daytime snack, NTS: nighttime snack; R: rhythmic, NR: not rhythmic

**
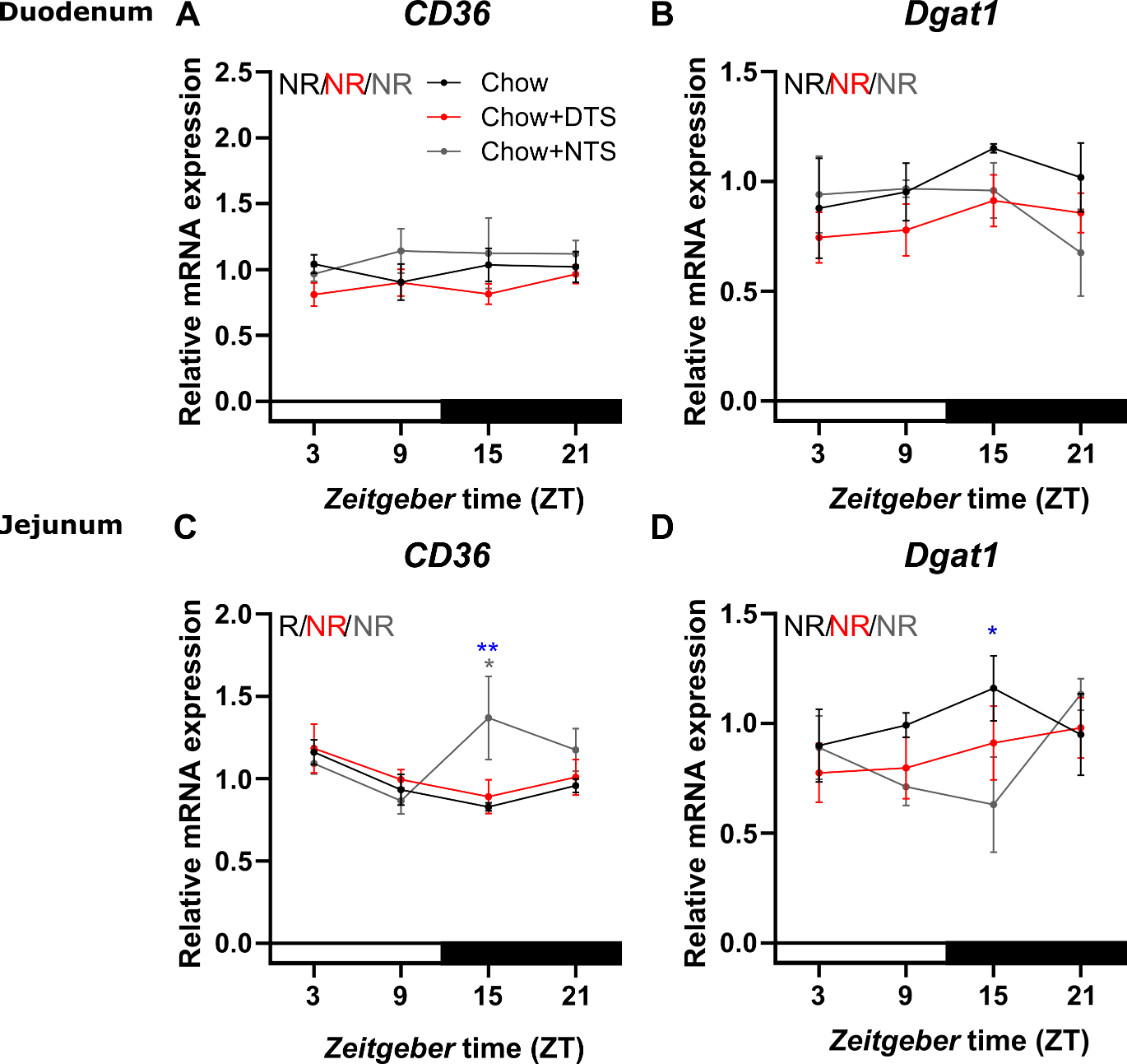
**

**A.7. Gene expression of *CD36* and *Dgat1* are mostly unaltered in the small intestine after snacking.** Gene expression of A,C) *CD36* and B,D) *Dgat1* in duodenum (A-B) and jejunum (C-D) after six weeks on snacking. Bonferroni post-hoc test * p<0.05, ** p<0.01, * chow vs. NTS, * DTS vs. NTS; 2-way ANOVA: time, group, interaction p>0.05. Data are shown as mean ± SEM; n=4-6 per ZT and group; DTS: daytime snack, NTS: nighttime snack; R: rhythmic, NR: not rhythmic

**
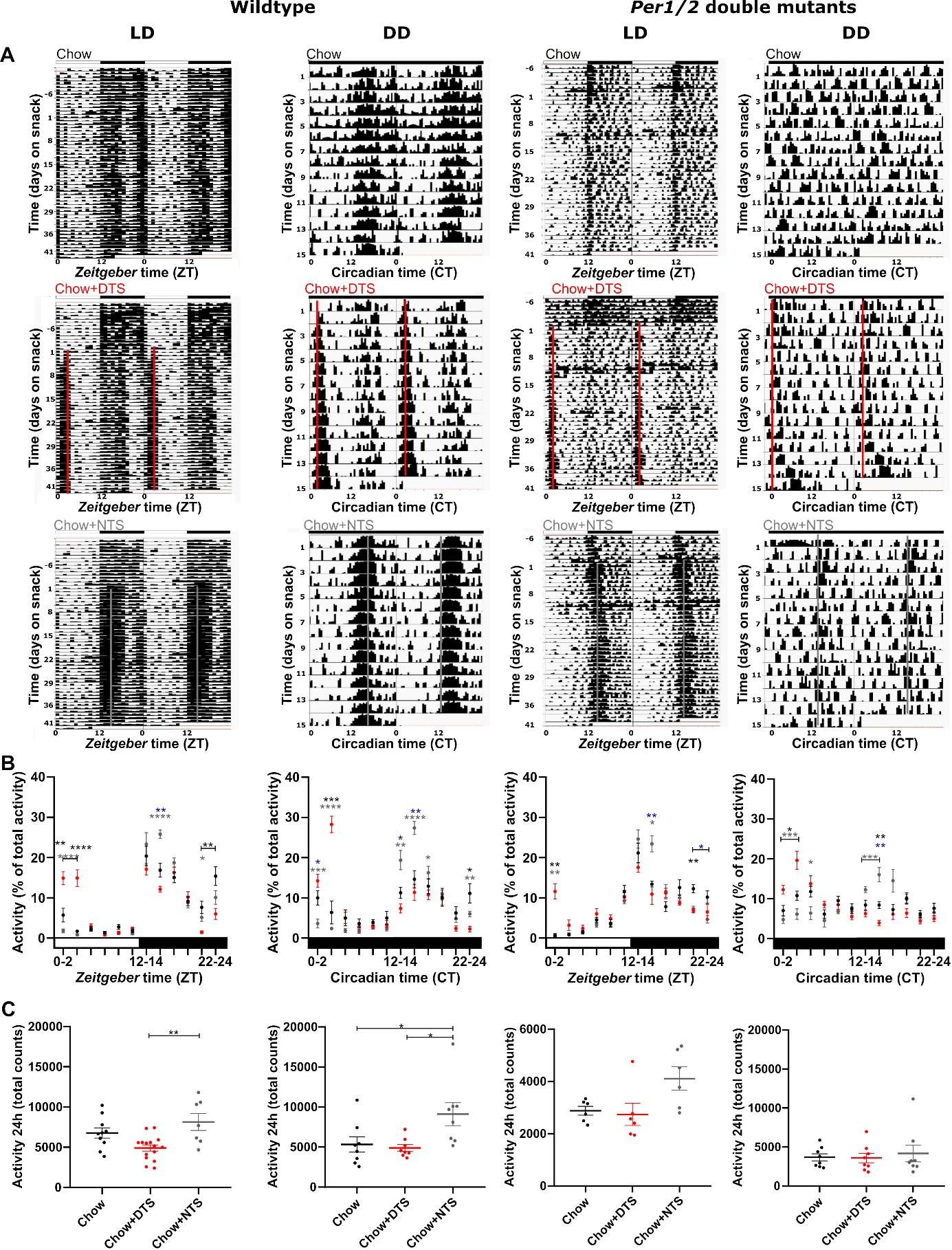
**

**A.8. Snacking alters locomotor activity rhythms in wildtype mice under LD and DD and in *Per1/2* double mutants under LD conditions.** A) Representative actograms of chow, DTS, and NTS (from top to bottom) wildtype mice housed in a regular 12h:12 light-dark cycle (LD) or in constant darkness (DD) and clock-deficient *Per1/2* double mutants in LD or DD. Actograms of wildtype mice in LD were replotted from Figure 4 for comparison. B) Daily locomotor activity profile as percentage of total activity and C) total 24h-activity measured by infrared detectors. From left to right: wildtype LD, wildtype DD, *Per1/2* LD, *Per1/2* DD. Bonferroni post-hoc test * p<0.05, ** p<0.01, *** p<0.001, **** p<0.0001, * chow vs. DTS, * chow vs. NTS, * DTS vs. NTS; B) 2-way ANOVA: time, interaction p<0.0001, group p>0.05. C) 1-way ANOVA: wildtype LD p<0.01, wildtype DD p<0.05, *Per1/2* LD p<0.05, *Per1/2* DD p>0.05. Data are shown as mean ± SEM; wildtype LD n=7-15, wildtype DD n=8, *Per1/2* LD n=6, *Per1/2* DD n=8; DTS: daytime snack, NTS: nighttime snack

**B. Detected lipids in the lipidomics screen.** Statistics (2-way ANOVAs and Bonferroni post-hoc tests) are added for each lipid.
